# Improving the spatiotemporal resolution of remotely sensed ET information for water management through Landsat, Sentinel-2, ECOSTRESS and VIIRS data fusion

**DOI:** 10.1007/s00271-022-00799-7

**Published:** 2022-05-21

**Authors:** Jie Xue, Martha C. Anderson, Feng Gao, Christopher Hain, Kyle R. Knipper, Yun Yang, William P. Kustas, Yang Yang, Nicolas Bambach, Andrew J. McElrone, Sebastian J. Castro, Joseph G. Alfieri, John H. Prueger, Lynn G. McKee, Lawrence E. Hipps, María del Mar Alsina

**Affiliations:** 1 Hydrology and Remote Sensing Laboratory, USDA-ARS, 10300 Baltimore Avenue, Beltsville, MD 20705 USA; 2 Earth Science Office, Marshall Space Flight Center, NASA, Huntsville, AL 35805 USA; 3 Sustainable Agricultural Water Systems Unit, USDA-ARS, Davis, CA 95616 USA; 4 Earth System Science Interdisciplinary Center, University of Maryland, College Park, MD 20742 USA; 5 Department of Land, Air and Water Resources, University of California, Davis, CA USA; 6 Crops Pathology and Genetics Research Unit, USDA-ARS, Davis, CA USA; 7 Department of Viticulture and Enology, University of California, Davis, CA USA; 8 National Laboratory for Agriculture and the Environment, USDA-ARS, Ames, IA USA; 9 Department of Plants Soils and Climate, Utah State University, Logan, UT USA; 10 E & J Gallo Winery, Viticulture, Chemistry and Enology, Modesto, CA USA

## Abstract

Robust information on consumptive water use (evapotranspiration, ET) derived from remote sensing can significantly benefit water decision-making in agriculture, informing irrigation schedules and water management plans over extended regions. To be of optimal utility for operational usage, these remote sensing ET data should be generated at the sub-field spatial resolution and daily-to-weekly timesteps commensurate with the scales of water management activities. However, current methods for field-scale ET retrieval based on thermal infrared (TIR) imaging, a valuable diagnostic of canopy stress and surface moisture status, are limited by the temporal revisit of available medium-resolution (100 m or finer) thermal satellite sensors. This study investigates the efficacy of a data fusion method for combining information from multiple medium-resolution sensors toward generating high spatiotemporal resolution ET products for water management. TIR data from Landsat and ECOSTRESS (both at ~ 100-m native resolution), and VIIRS (375-m native) are sharpened to a common 30-m grid using surface reflectance data from the Harmonized Landsat-Sentinel dataset. Periodic 30-m ET retrievals from these combined thermal data sources are fused with daily retrievals from unsharpened VIIRS to generate daily, 30-m ET image timeseries. The accuracy of this mapping method is tested over several irrigated cropping systems in the Central Valley of California in comparison with flux tower observations, including measurements over irrigated vineyards collected in the GRAPEX campaign. Results demonstrate the operational value added by the augmented TIR sensor suite compared to Landsat alone, in terms of capturing daily ET variability and reduced latency for real-time applications. The method also provides means for incorporating new sources of imaging from future planned thermal missions, further improving our ability to map rapid changes in crop water use at field scales.

## Introduction

Agriculture is impacted by many environmental issues, including climate change, and degradation of land and freshwater (Foley et al. 2011; Hall et al. 2008). In agricultural regions, water availability is one of the most important factors determining crop quality and production. In recent years, there have been increasing demands on global freshwater resources for food production to support the world’s growing population. At the same time, freshwater availability has become increasingly limited in some production regions due to changing atmospheric conditions, aquifer overdrafts, extended droughts, and increased competing uses (Ficklin and Novick 2017; Richey et al. 2015). To develop sustainable agricultural systems, there is a critical need for accurate assessments of water use and increased water-use efficiency. Agricultural water management requires detailed information about crop water use and soil moisture status at field scales, which is conveyed by daily to seasonal estimates of evapotranspiration (ET)—the main form of agricultural water loss.

Remote sensing data are well suited to providing accurate estimates of ET at the spatial scales required for agricultural management, with low demands for ancillary information and at low cost (Anderson et al. 2012). Over the past few decades, a number of remotely sensed surface energy balance (SEB) approaches have been developed for mapping ET that exploit the land surface temperature (LST) derived from satellite thermal bands as a proxy indicator of water status (Allen et al. 2007; Anderson et al. 1997, 2004, 2007; Bastiaanssen et al. 1998; Norman et al. 2003). In particular, thermal-based energy balance approaches are sensitive to rapid changes in moisture status and, therefore, capable of effectively capturing vegetation water use changes. This is due to the fact that canopy temperature tends to change faster under stress than many other canopy biophysical properties reflected in optical imagery (Moran 2004). In addition, thermal signals are also sensitive to soil moisture changes under low canopy cover (e.g., early season irrigation). A suite of optical and thermal ET modeling systems form the basis for OpenET (Melton et al. 2021)—a powerful tool for improved agricultural water management providing field-scale ET estimates across the western United States.

In addition, anomalies in ET (i.e., deviations of current ET from normal ET, as averaged over a relatively long period) show great potential for identifying agricultural drought onset and impacts. The Evaporative Stress Index (ESI), defined as temporal anomalies in the ratio of actual-to-reference ET (*f*_RET_), has proven to be a good leading indicator of stress, particularly in rapid onset, or “flash” drought events (Anderson et al. 2013, 2015, 2016; Otkin et al. 2013). Regional ESI generated at 4–10 km scale over the U.S. using thermal data from geostationary satellites has been demonstrated its utility as an agricultural drought indicator (Otkin et al. 2018, 2019). However, the relatively coarse spatial resolution of these products in some cases may not adequately separate the stress responses of individual land-cover types (e.g., crop vs. forest) and cannot provide effective guidance for field-scale irrigation management. More recently, attempts have been made to generate ESI at crop stand scale by using high spatial-resolution data (30–100 m) from Landsat (Knipper et al. 2019b; Yang et al. 2020, 2021a, 2018).

To date, Landsat has been considered a gold standard for field-scale ET monitoring and stress detection, providing data in the visible, near-infrared, shortwave infrared (VSWIR) and thermal infrared (TIR) bands required for SEB modeling, all co-collected from a single platform and with excellent calibration and registration (Anderson et al. 2012). However, the relatively low revisit frequency of Landsat imagery (i.e., 16 days for a single system; Table 1) constrains full reconstruction of daily and seasonal water use dynamics at field scales, particularly during periods of rapid change including green-up, harvest, intensive management, and flash drought. Previous studies have investigated the integration of other thermal data sources in addition to Landsat to improve temporal sampling. For example, the Moderate Resolution Imaging Spectroradiometer (MODIS) provides imaging for ET retrieval at coarser scales (~ 500 m) on a near-daily basis (Table 1). Fusing high-spatial/low-temporal resolution Landsat ET with low-spatial/high-temporal resolution MODIS ET timeseries (Gao et al., 2006; hereafter referred to as “Landsat-only fusion”) has proven effective in generating ET timeseries with both high spatial and temporal resolution over a range of land cover types (Anderson et al. 2018; Cammalleri et al. 2013; Knipper et al. 2020; Semmens et al. 2016; Sun et al. 2017; Yang et al. 2017). Still, the accuracy of fused timeseries strongly depends on the frequency of the high-resolution ET retrievals anchoring the fusion process; therefore, other studies have investigated the utility of including other medium resolution thermal data sources in addition to Landsat. For example, Anderson et al. (2021) incorporated ECOsystem Spaceborne Thermal Radiometer Experiment on Space Station (ECOSTRESS) as an additional Landsat-like TIR data source. Xue et al. (2021) tested using TIR data from the Visible Infrared Imaging Radiometer Suite (VIIRS) I–5 band together with VSWIR data from Sentinel-2 (S2; Table 1) for augmenting Landsat ET timeseries. Both studies have demonstrated the value of additional temporal sampling complementing Landsat in improved ET monitoring.

**Table 1:** Characteristics of satellite data used in this study

Platform/sensor	Launch date	Equatorial crossing time	Spatial resolution		Temporal resolution
			SR bands (m)	TIR bands	
Landsat 8	Feb. 11, 2013	10:00 a.m.	30	100	16 day
Sentinel-2A	Jun. 23, 2015	10:30 a.m.	10–20	–	10 day
Sentinel-2B	Mar. 7, 2017	10:30 a.m.	10–20 m	–	10 day
ECOSTRESS	Jun. 29, 2018	–	–	~ 70	1–5 day
VIIRS	Oct. 28, 2011	1:30 p.m.	(I bands) 375	375	~ daily
			(M bands) 750	750	
MODIS	(Terra) Dec. 18, 1999	10:30 a.m.	250–500	1000	A few times per day
	(Aqua) May 5, 2002	1:30 p.m.			

In this study, we propose to combine ET retrievals from ECOSTRESS and VIIRS I-5 with Landsat imaging to provide even more frequent medium-resolution ET sampling capabilities (referred to as Landsat + ECOSTRESS + VIIRS_*I*_/S2 fusion). We evaluated the improvement in fusion results over Landsat-only fusion in capturing disturbance events (e.g., rainfall, flash drought, green-up, and harvest) in which the resulting changes in crop water use are transient and not recorded in available Landsat scenes. These two fusion approaches (Landsat-only and Landsat + ECOSTRESS + VIIRS_*I*_/S2 fusion) were used to create 30-m daily ET datacubes for the period of 2018–2020 over three domains in California, U.S., sampling a broad range of climate and water management practices. The accuracy of modeled ET timeseries is evaluated in comparison with long-term flux tower measurements collected over these study domains. We also propose VIIRS M-band products to replace MODIS (soon to be extinct) as the new ET fusion backbone and test its feasibility. The goal is to lay a new foundation for future enhancements to projects including OpenET and ET-based drought monitoring, to demonstrate the value of more frequent thermal imaging, and to suggest methods for multi-source integration looking forward to the future medium-resolution TIR satellite missions. We also investigate the effectiveness of ET anomalies in capturing the impacts of crop management practices and stress in water use at both daily and monthly time steps and sub-field scales. The overall aim of this work was to evaluate the capabilities of the multi-source ET modelling framework for reconstructing daily and seasonal ET dynamics at field scale.

## Study area

**Fig. 1:**
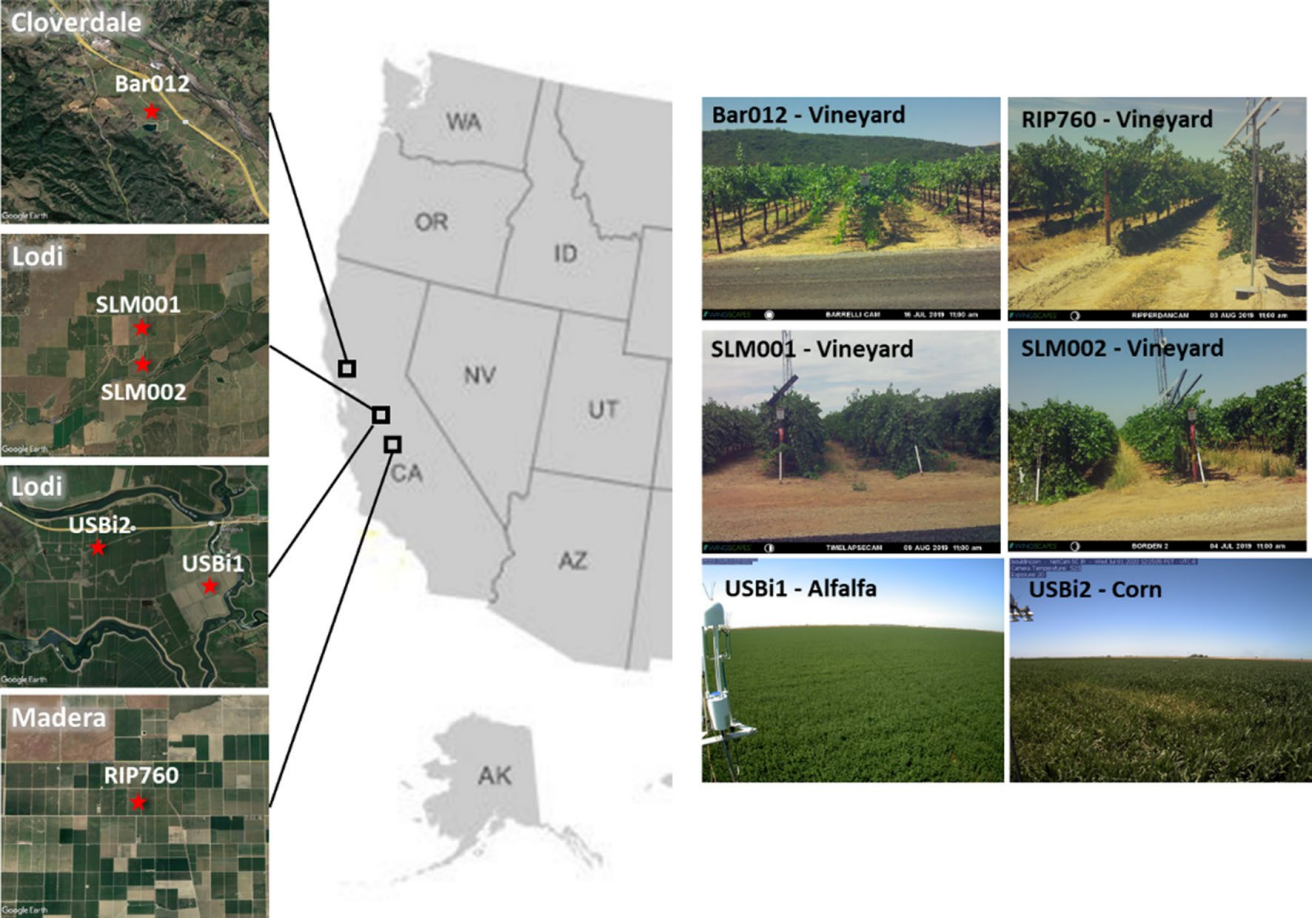
Three study domains (Cloverdale, Lodi and Madera) in California, U.S. (center column). Flux tower locations are indicated with red stars on the Google Earth true color images (left column). Site photographs to the right are from PhenoCams installed at each flux site (colour figure online)

The study domain, as illustrated in Fig. 1, includes three grape-growing regions in California (CA), USA, that were sampled as part of the USDA-ARS Grape Remote Sensing Atmospheric Profile and Evapotranspiration eXperiment (GRAPEX) (Kustas et al. 2018). The three vineyard sites are near Cloverdale, CA (site name BAR); near Lodi, CA (site name SLM); and near Madera, CA (site name RIP). Together they sample a north–south gradient in climatic conditions due to variations in precipitation, temperature and humidity, along with different grape varieties, row-orientations, and soil types. The SLM and RIP sites are located in the Central Valley, while the northern BAR site is in the Sonoma Valley area. The climate changes from north coastal region to the southern Central Valley with decreasing precipitation and increasing air temperature. The BAR site has cooler and moister air conditions, while the SLM and RIP sites experience a warm Mediterranean climate characterized by warm days and cool nights (Knipper et al. 2020). The primary land cover type of the three sites is irrigated vineyards, surrounded with irrigated orchards and pastures, and unirrigated grasslands (Fig. 1). In addition to the GRAPEX sites, flux sites on Bouldin Island, CA sampling irrigated alfalfa (USBi1) and corn (USBi2) fields were also included in our analyses to test the utility of integration of data from multiple Landsat-like platforms.

The Central Valley aquifer system that supports irrigation in this agricultural production area is extremely stressed with an annual depletion rate of −20 mm year^−1^ from groundwater for the period 2003–2010 and even higher during drought years (Famiglietti et al. 2011; Richey et al. 2015). The extensive groundwater extraction has resulted in land subsidence in many areas of the Central Valley over the last decades, partially related to regions where non-permanent crops and pastures were converted to long-term crops such as vineyards with a relatively inflexible demand for water (Faunt and Sneed 2015; Faunt et al. 2016). Possible strategies to deal with increasingly limited water resources include more efficient irrigation methods or converting to crops with lower water needs (Anderson et al. 2018). In addition, an improved understanding of the water use changes associated with these management practices is urgently needed. Previous studies (Knipper et al. 2019b; Semmens et al. 2016) focusing on these regions have demonstrated that the 16-day revisit of Landsat 8 was not enough to capture the vineyard response to stress. Other studies showed Landsat sampling was even more insufficient for crops like alfalfa which have higher ET temporal dynamics due to monthly cuttings, and pose a significant challenge to ET modeling (Anderson et al. 2021; Xue et al. 2021).

In this study, we focus on a 3-year period from 2018 to 2020 (2020 is a drought year), after the severe drought (2012–2017) in California. This period was characterized by a variety of meteorological conditions and soil water availability, with varying cropping and irrigation strategies in the Central Valley. A modeling domain of 90 × 90 km^2^ centered on the target flux tower sites was used. Detailed information about each flux tower site is provided in “Study sites and flux tower measurements”.

## Methods

**Fig. 2:**
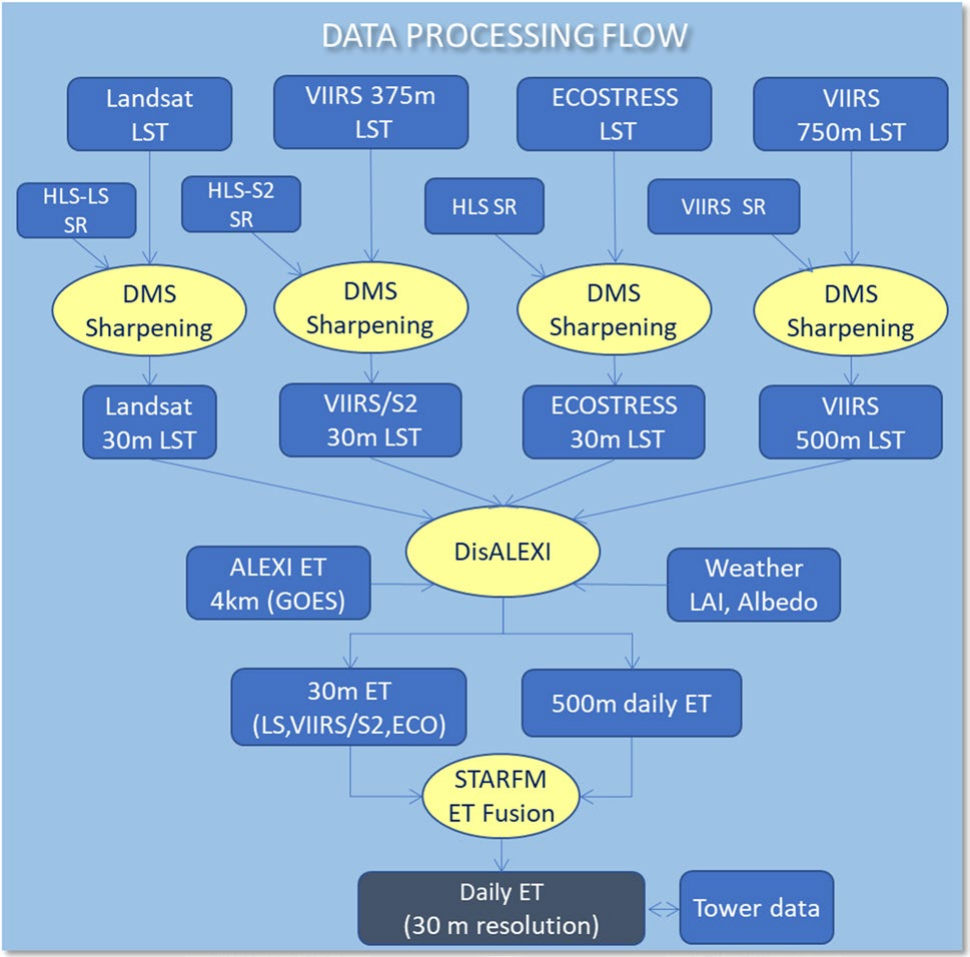
Schematic diagram illustrating the flow of the multi-source ET modelling system

In this study we use a multi-scale ET modelling system to generate various ET data with inputs from multi-source TIR and SR data. A Data Mining Sharpener (DMS) approach is applied to sharpen raw LST inputs for DisALEXI to finer resolution. The output ET data on overpass dates are fused to generate 30-m daily ET time series using the Spatial and Temporal Adaptive Reflectance Fusion Model (STARFM). A schematic diagram illustrating the flow of ET processing is shown in Fig. 2, with each component of the system explained in greater detail below and in “Data and analyses”.

### Multiscale ET modelling framework

The multi-scale ET modelling framework used here is based on the regional Atmosphere-Land Exchange Inverse (ALEXI) model (Anderson et al. 2007) and the associated disaggregation scheme DisALEXI (Anderson et al. 2004; Norman et al. 2003), which are built upon the Two Source Energy Balance (TSEB) model (Kustas and Norman 1997; Norman et al. 1995). The TSEB estimates the surface energy balance and radiometric temperature separately for the soil and canopy components of each model pixel, partitioned based on the local fractional vegetation cover or leaf area index (LAI). To reduce the uncertainty in remote sensing thermal retrievals, in the regional ALEXI model, the TSEB is used in a time-differencing mode by coupling with a slab model of the atmospheric boundary layer to morning thermal observations collected by geostationary satellites at relatively coarse spatial resolution (roughly 3–10 km). The DisALEXI component of the framework spatially disaggregates ALEXI daily fluxes by applying TSEB to finer-resolution thermal data from polar orbiting satellites. In DisALEXI, the air temperature is iteratively tuned at the ALEXI pixel scale to enforce consistency between ALEXI and reaggregated DisALEXI fluxes. This normalization process also ensures the consistency between DisALEXI fluxes from different sensors with thermal data acquired at different times of day, a key factor in successful multi-source data fusion. For more ALEXI/DisALEXI modelling details, the reader is referred to Cammalleri et al. (2013) and Sun et al. (2017).

### Multi-source data fusion scheme

#### Thermal data sharpening

In multi-spectral satellite imaging systems, the spatial resolution of thermal infrared (TIR) data is typically coarser than that of surface reflectance (SR) data from the same platform. However, the TSEB land-surface representation in ALEX/DisALEXI requires physical and spatial consistency between the LST and LAI inputs derived from TIR and SR imagery, respectively. To achieve resolution consistency between bands and platforms, the DMS (Gao et al. 2012b; Xue et al. 2020) approach was used in this study to sharpen Landsat-like LST imagery (i.e., Landsat, ECOSTRESS and VIIRS I–5 band) from native resolution (i.e., 100, ~ 70 and 375 m, respectively) to 30 m and MODIS-like LST (i.e., MODIS and VIIRS M-bands) from 1 km and 750 m, respectively, to 500 m. DMS is a machine-learning algorithm that builds an ensemble of cubist regression trees between SR and LST data at the coarse resolution of the TIR bands and then uses these statistical relationships to predict LST at the finer resolution of the SR bands. To conserve energy at coarse resolution, the residuals between the actual LST and modeled LST at coarse resolution are interpolated and applied to the fine resolution LST image. VSWIR bands were used to sharpen Landsat-like LST images, while NDVI band was used for MODIS-like LST sharpening. It should be noted that the purpose of MODIS-like LST sharpening is mainly to reduce the bowtie effect due to off-nadir pixel smearing (Gómez-Landesa et al. 2004). In this case, DMS was run using NDVI, similar to the localized TsHARP approach which was reported effective in prior studies (Cammalleri et al. 2014; Kustas et al. 2003; Semmens et al. 2016; Sun et al. 2017). Note that in addition to the enhanced spatial resolution of LST, thermal sharpening also improves the spatial consistency between LST and SR (including LAI, NDVI, and albedo) inputs for the DisALEXI model, which helps relieve spatial misalignment in data from different satellite platforms or LST data noise that may come from an imperfect atmospheric correction. For instance, a modified DMS approach (Xue et al. 2020) with a relaxed scale of energy conservation was successfully applied to ECOSTRESS and VIIRS LST sharpening to 30 m.

#### ET data fusion

Periodic Landsat-like and daily MODIS-like ET retrievals were integrated into a single 30-m/daily timeseries using the STARFM algorithm, which provides a feasible way for fusing remote sensing data from different satellite platforms (Gao et al. 2006). STARFM combines high spatial information from Landsat-like sensors and high temporal information from MODIS-like sensors to generate data with high resolution in both time and space. The STARFM approach takes advantage of the continuous phenology changes observed by MODIS-like timeseries to benefit the prediction between Landsat-like overpasses. In STARFM, one or more existing Landsat/MODIS image-pairs collected on the same date are used to predict Landsat-like images on other MODIS observation dates when Landsat data are not available. STARFM employs a weighting function describing the spectral, spatial and temporal relationship between existing Landsat/MODIS image-pairs to downscale MODIS images on prediction dates. One-pair STARFM uses a single image pair to predict images on dates surrounding the pair date, while two-pair STARFM uses two nearest image pairs bracketing the prediction date. The application of STARFM in ET fusion has been widely investigated over various land cover types and most of these works used one-pair STARFM due to its simplicity and flexibility in near-real-time applications (Anderson et al. 2021; Knipper et al. 2019a; Yang et al. 2017). While most previous ET fusion studies have used the one-pair fusion method, in this study, we used the dual-pair method described by Yang et al. (2022) as it has been found to generate smoother ET timeseries with smaller errors in highly dynamic agricultural systems.

In this study, two categories of ET data with different spatial and temporal resolutions were directly retrieved using DisALEXI. One is high-spatial low-temporal resolution (30 m; periodic) Landsat-like ET including Landsat, ECOSTRESS and VIIRS_*I*_/S2. The other is low-spatial high-temporal resolution (500 m; ~ daily) MODIS-like ET including MODIS and VIIRS_*M*_. The two sets of ET data were fused using STARFM, combining each dataset’s strength to generate ET datacubes at 30-m resolution and daily time scale. We tested two fusion scenarios: (1) 30-m Landsat ET retrievals with 500-m VIIRS_*M*_ daily ET; (2) 30-m Landsat + ECOSTRESS + VIIRS_*I*_/S2 ET retrievals with 500-m VIIRS_*M*_ daily ET. The two fused datacubes were compared with flux tower observations to evaluate the value added by the additional temporal sampling from ECOSTRESS and VIIRS_*I*_/S2. The two fusion schemes were also applied in simulated real-time scenarios based on one-pair STARFM. In addition, for the first scenario, we also fused Landsat ET estimates with 500-m MODIS ET, which was used as a benchmark to evaluate the performance of 500-m VIIRS_*M*_ ET as the new post-MODIS era fusion backbone.

### Evaporative stress index (ESI)

ESI is a remote sensing-based agricultural drought index, defined in terms of anomalies in *f*_RET_ relative to a long-term mean. An advantage of ESI is it does not require information about the soil moisture status or rainfall, but rather diagnoses vegetation stress via impacts of elevated canopy temperature on the ET retrieval (Anderson et al. 2011, 2007). Normalizing by reference ET better focuses on the soil moisture signal, reducing the impact of seasonal variations of available energy and evaporative demand on actual ET. Here we use reference ET for a short grass under well-watered conditions calculated based on the FAO-56 Penman–Monteith formulation (Allen et al. 1998). ESI has been demonstrated to be a valuable early warning indicator of stress at both regional scale (Anderson et al. 2013; Otkin et al. 2013, 2019) and sub-field scale (Yang et al. 2021a, 2018).

At regional-to-continental scales, ESI is typically calculated as a standardized anomaly relative to long-term baseline conditions. First, *f*_RET_ composites are generated within 4-, 8- and 12-week windows moving with a 7-day time step to investigate the drought impacts at different timescales. Then standardized anomalies in the *f*_RET_ composites are computed to highlight differences in moisture conditions between years, normalizing each composite interval to a mean of 0 and standard deviation of 1 using mean and standard deviation computed for that time window over a multi-decade baseline period. Currently, routine ESI products are generated over the continental United States (CONUS) and globally based on ALEXI ET, and have shown comparable accuracy with other standard drought indicators (Anderson et al. 2015).

For sub-field scale stress analyses, a non-standardized *f*_RET_ anomaly (named Δ* f*_RET_) has generally been used due to the shorter time-frame associated with these analyses (Knipper et al. 2019a; Yang et al. 2021a, 2018, 2021b). This study investigates the utility of both fused *f*_RET_ timeseries and *f*_RET_ anomaly (named Δ*f*_RET_) for stress detection, computed for the 3-year study period at both daily and monthly average scales.

## Data and analyses

### Study sites and flux tower measurements

Each study domain discussed in “Study area” contains one or more flux towers equipped with eddy covariance (EC) and meteorological sensors measuring surface fluxes and micrometeorological data. Data collected at six towers are used to evaluate model performance, model crop biomass changes, and detect vine stress (Table 2). This includes four GRAPEX towers in irrigated vineyards, all equipped with similar instrumentation (Alfieri et al. 2019; Knipper et al. 2020). The BAR012 tower is located in the Cloverdale domain. The grapes in this block are Cabernet Sauvignon and planted in 2010 with an area of 10 ha. The RIP760 tower is sited in the Madera domain, with 31-ha Chardonnay grapes planted in 2010. Measurements of surface energy fluxes and meteorological data at SLM001 and SLM002 sites began in 2013 and in 2017 for BAR012 and RIP760, respectively.

**Table 2:** Characteristics of flux towers used in this study

Domain	Tower	Land cover	Vine variety	Year planted	Latitude	Longitude
Lodi	SLM001	vineyard	Pinot Noir/Cabernet Sauvignon	2009/2020	38.289	− 121.118
Lodi	SLM002	vineyard	Pinot Noir/Merlot	2011/2020	38.280	− 121.118
Lodi	USBi1	alfalfa	–	2016	38.099	− 121.499
Lodi	USBi2	corn	–	2017	38.109	− 121.535
Cloverdale	BAR012	vineyard	Cabernet Sauvignon	2010	38.751	− 122.975
Madera	RIP760	vineyard	Chardonnay	2010	36.839	− 120.210

Flux towers SLM001 and SLM002 are within the Lodi domain. Both vineyard blocks are planted with Pinot noir vines since 2009 and 2011 with an area of 35 ha and 21 ha, respectively. The Lodi domain also contains two AmeriFlux towers (https://ameriflux.lbl.gov/), USBi1 and USBi2, located in the California Delta regions (Bouldin Island). The two towers are situated in irrigated fields with USBi1 in alfalfa since 2016 and USBi2 in corn since 2017 (Anderson et al. 2021; Eichelmann et al. 2018). The observed fluxes from the two towers were also used in this study to compare with modeled fluxes.

Model results are compared to flux measurements from EC systems as observed, and with a correction for energy balance closure error, which is reported on the order of 10–30% (Allen et al. 2011). Following previous studies (Anderson et al. 2021; Xue et al. 2021), a residual correction approach was used at most sites, assigning the energy budget residual to the daily latent heat flux. At SLM001 and SLM002, a modified residual correction was used to minimize impacts of nighttime shifts in wind patterns from off-field (Anderson et al., 2018). At each site, closure corrections were limited to 30% for days with closure errors exceeding 30% of unclosed measurement. Modeled daily fluxes were compared with both unclosed and closed tower observations under the assumption that these likely bound the true flux value and provide a metric of uncertainty in the flux observations (Twine et al. 2000).

### DisALEXI model inputs

In the DisALEXI model component, CONUS-wide ALEXI daily ET at 4-km scale (Anderson et al. 2007) was used as the baseline for disaggregation. Other major inputs to DisALEXI include remote sensing data (LST, LAI, and albedo) and meteorological data (air temperature, atmospheric pressure, vapor pressure, wind speed, and insolation). Moderate ET at 500-m scale was obtained by applying DisALEXI to MODIS-like data and used as the fusion backbone. Three sets of sub-field ET at 30-m resolution were obtained by applying DisALEXI at Landsat-like scale. In this section, we provide a brief overview of the key inputs for each ET disaggregation scheme.

#### DisALEXI-MODIS and DisALEXI-VIIRS_*M*_

MODIS ET estimates at 500-m resolution were generated with DisALEXI (DisALEXI-MODIS) by disaggregating ALEXI 4-km ET using MODIS LST and VSWIR products. Three products from Collection 6 were used: LST (MOD11_L2; (Wan et al. 2015)), LAI (MCD15A3H; (Myneni et al. 2015)) and albedo (MCD43A3; (Wang et al. 2018)). The LAI and albedo products are at 500-m resolution, and the LST product provided at 1-km resolution was sharpened to 500 m using the DMS approach described in “Thermal data sharpening”.

The VIIRS instrument follows the legacy of MODIS and collects imagery at two spatial resolutions: 750 m (M-bands) and 375 m (I-bands). In this study, we also generated 500-m ET estimates using VIIRS 750-m LST data with DisALEXI (DisALEXI-VIIRS_*M*_) and evaluated its performance in comparison with DisALEXI-MODIS, which has been used as the fusion backbone in all ET STARFM fusion experiments to date. DisALEXI-VIIRS_*M*_ used instantaneous LST and emissivity version-1 swath product (VNP21; (Hulley and Hook 2018)) constructed at daily timesteps and 750-m spatial resolution, and sharpened to 500 m using DMS. Other inputs to DisALEXI-VIIRS_*M*_ include the 8-day 500-m LAI composite (VNP15A2H; (Myneni and Knyazikhin 2018)), 16-day 500-m vegetation indices composite (VNP13A1; (Didan and Barreto 2018)), and 1-km daily albedo (VNP43MA3; (Liu et al. 2017)) resampled to 500-m pixel scale.

Both the near-daily DisALEXI-MODIS and DisALEXI-VIIRS_*M*_ ET retrievals were gap-filled and smoothed to generate daily 500-m ET datacubes (Sun et al. 2017).

#### DisALEXI-Landsat, DisALEXI-VIIRS_*I*_/S2 and DisALEXI-ECOSTRESS

ALEXI ET fields were also disaggregated to 30 m with three sets of inputs from Landsat 8, Sentinel-2 plus VIIRS 375-m LST, ECOSTRESS plus harmonized Landsat and Sentinel-2 (HLS). The Landsat 8 and Sentinel-2 data used in this study come from NASA’s HLS v1.4 dataset (https://hls.gsfc.nasa.gov/; L30 and S30 products) which provides a consistent surface reflectance data product from Landsat and Sentinel-2 (Claverie et al. 2018).

For Landsat disaggregation (DisALEXI-Landsat), Landsat 7 was not used in this study due to gap stripes resulting from the failure of the scan-line corrector. Instead, Landsat 8 SR and TIR data from HLS dataset (L30) were used to generate 30-m ET retrievals at Landsat overpass dates. TIR data were atmospherically corrected using MODTRAN (Cook et al. 2014) and then sharpened using DMS from 100 to 30 m for consistency with the SR data. 30-m LAI was estimated by downscaling the MODIS 500-m LAI product using a regression tree approach (Gao et al. 2012a) and Landsat SR data. 30-m albedo was derived based on a narrowband to broadband conversion formulae developed for Landsat data (Liang 2001).

The combined 3- to 4-day revisit of HLS makes it attractive for ET mapping at sub-field scales; however, S2 does not collect the TIR data required for ET modelling. VIIRS I-5 band provides TIR data at 375 m and near-daily temporal revisit. The combination of SR inputs from S2 and TIR from VIIRS has been successfully applied for 30-m ET disaggregation (namely DisALEXI-VIIRS_*I*_/S2) on S2 overpass dates (Xue et al. 2021). Given that the S2 SR data from HLS (S30) are co-registered and spectrally adjusted with respect to Landsat 8 (L30) and resampled to 30-m resolution, S2 LAI and albedo model inputs were constructed similarly to Landsat. VIIRS I-5 brightness temperatures were downloaded from NASA LANCE and atmospherically corrected using a single channel inversion (Price 1983). The VIIRS LST data were then sharpened to 30 m by the modified DMS approach (Xue et al. 2020) using S2 SR data as inputs. As demonstrated by Xue et al. (2021), 30-m ET retrievals from DisALEXI-VIIRS_*I*_/S2 show comparable accuracy with DisALEXI-Landsat, with performance slightly affected with larger view angles. As suggested by Xue et al. (2021), we removed VIIRS LST data with view angle greater than 52° in this study.

The final set of 30-m ET was ECOSTRESS-based (namely DisALEXI-ECOSTRESS). ECOSTRESS is a thermal-only instrument, so the SR inputs for ECOSTRESS disaggregation have to be obtained from other satellites. In a previous study, Landsat 7 + 8 SR data collected closest in time to the target ECOSTRESS LST date were used for ET disaggregation (Anderson et al. 2021). Anderson et al. (2021) found that the temporal separation between SR and TIR inputs impacts the accuracy of ET retrievals. In this study, we use HLS SR data instead to generate LAI and albedo inputs because the 3-to 4-day temporal frequency of HLS data are higher than combined Landsat 7 and 8 (8 days). ECOSTRESS level-2 LST and emissivity (ECO2LSTE) and cloud mask swath products (ECO2CLOUD) were downloaded from USGS Land Processes Distributed Active Archive Center (LP DAAC) and gridded to the geographical coordinates. The ECOSTRESS LST data were resampled to a 30-m UTM grid to match with HLS data and sharpened to 30 m using the modified DMS approach (Xue et al. 2020), which was found critical to relieving spatial misregistration and enhancing the consistency in SR and TIR inputs to DisALEXI modelling. Anderson et al. (2021) found that the ECOSTRESS LST data at view angles greater than 20° resulted in poor sharpening results. Therefore, we carefully screened the ECOSTRESS LST data exceeding 20° before use and removed those with poor performance from further analysis.

Spatial gaps in the generated three sets of 30-m ET images due to cloud cover were gap-filled using the technique developed by Yang et al. (2017). For days with more than one 30-m ET retrieval, the order of priority for inclusion in the fused timeseries is DisALEXI-Landsat, followed by DisALEXI-VIIRS_*I*_/S2, and DisALEXI-ECOSTRESS since DisALEXI-Landsat typically has the best accuracy. For more details about DisALEXI-Landsat, DisALEXI-ECOSTRESS and DisALEXI-VIIRS_*I*_/S2 model inputs, the reader is referred to see Sun et al. (2017), Anderson et al. (2021) and Xue et al. (2021).

### U.S. Drought monitor time series

The U.S. Drought Monitor (USDM) is developed through the integration of a variety of drought indicators including precipitation, soil moisture, groundwater storage, and local reports from field observers. USDM timeseries data for the State of California and the Sacramento County (Lodi) from 2018 to 2020 were extracted from the National Drought Mitigation Center at the University of Nebraska-Lincoln (https://droughtmonitor.unl.edu/DmData/TimeSeries.aspx) and used to demonstrate the crop water status under various rainfall and water management practices. USDM classifies drought into five classes and maps them with numerical values: *D*0 = 0 (abnormally dry), *D*1 = 1 (moderate drought), *D*2 = 2 (severe drought), *D*3 = 3 (extreme drought), and *D*4 = 4 (exceptional drought). The timeseries show the temporal evolution of percent area covered by each class within a county or state.

### Model evaluation configurations

In this study, the accuracy of ET retrievals was evaluated in five stages. We first evaluate the relative performance of 500-m/daily ET estimates from DisALEXI-VIIRS_*M*_ and DisALEXI-MODIS, serving as the daily backbone in the STARFM fusion process. This is a necessary first step as we transition away from retrievals based on MODIS, which is nearing the end of life. We then compare 30-m ET estimates derived from, ECOSTRESS and VIIRS_*I*_/S2 on satellite overpass dates with Landsat retrievals as a reference, and with the flux tower observations. To compare with tower observations, modelled 30-m ET were averaged over a 3 × 3 window (90 × 90 m) centered on the tower location (Anderson et al. 2021; Xue et al. 2021), approximating the flux source area which is typically on the order of 100 m, depending on the tower height and surface conditions (Li et al. 2008). We note that more sophisticated flux footprint models (e.g., Kljun et al. 2015) may provide a more accurate comparison. We moved the center of extraction by a few pixels to avoid edge effects for towers located at field edges. For 500-m ET, the pixel containing the towers is selected to compare with fluxes measurements. Next, daily 30-m ET datasets generated from Landsat-only fusion and Landsat + ECOSTRESS + VIIRS_*I*_/S2 fusion are compared to investigate the added value of increased temporal sampling frequency by the additional medium-resolution ET samples. In this case, STARFM fusion is performed in both retrospective (two-pair) and real-time (one-pair) modes to explore the benefits of the extra temporal sampling for real-time applications. Finally, the capabilities of the fused daily ET timeseries in capturing rapid changing crop conditions are assessed, including changes due to crop management practices and flash drought.

Statistical comparisons with flux tower observations include mean absolute error (MAE), root mean square error (RMSE), mean bias error (MBE), and relative error (RE = MAE/ <*O*>) where <*O*> is the mean observed flux. The MAE is a better indicator of model performance, while RMSE is more sensitive to isolated outliers (Willmott and Matsuura 2005)—for the fused timeseries, both metrics are provided for comparison.

## Results

### Evaluation of DisALEXI-VIIRS_*M*_ 500-m ET

**Fig. 3:**
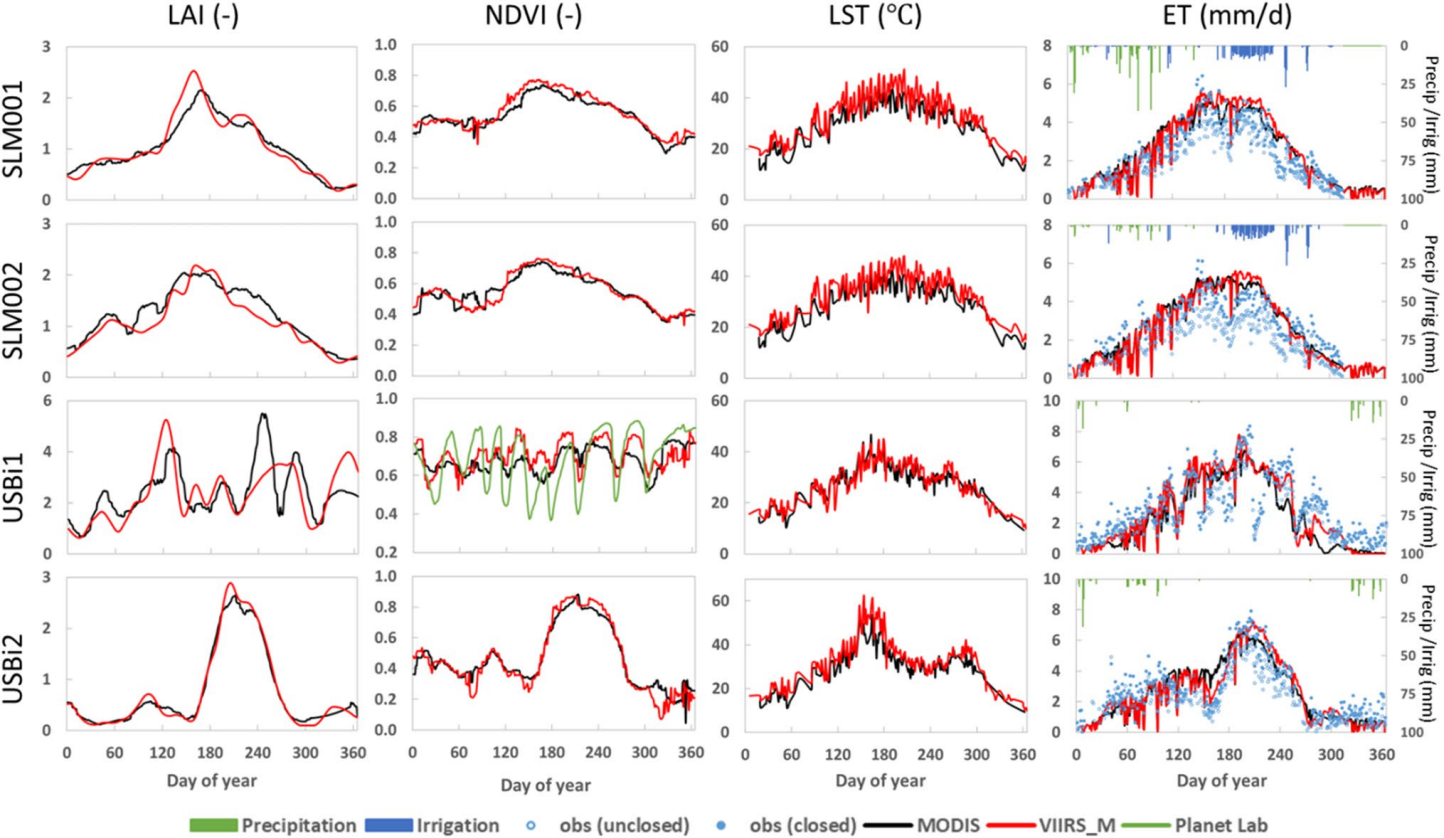
Time series of modeled MODIS (black line) and VIIRS_*M*_ (red line) daily LAI, NDVI, LST and ET, and the Planet Labs NDVI (green line) for SLM001, SLM002, USBi1 and USBi2 sites in 2018. Blue circles are closed (solid) and unclosed (open) ET observations at flux tower sites. Green and blue vertical bars indicate precipitation and irrigation data, respectively. Note that irrigation data for USBi1 and USBi2 sites are not available (colour figure online)

To evaluate the relative performance of the VIIRS_*M*_ and MODIS 500-m ET timeseries, modeled ET timeseries of 2018 from both DisALEXI-MODIS and DisALEXI-VIIRS_*M*_ were compared with flux tower observations over the four sites in the Lodi domain (SLM001, SLM002, USBi1, and USBi2) (Fig. 3). Note that the 500-m pixel scale is much coarser than the footprint of the EC tower in each case (~ 100 m), so we do not expect perfect agreement between models and observations at this scale. Still, the comparison can provide some insights into the relative biases in comparison with ET observations. In addition, Fig. 3 also provides the comparisons of inputs to DisALEXI modeling including LAI, NDVI, and LST timeseries. For NDVI at the USBi1 site, we additionally have NDVI at 5-m resolution from Planet Labs in 2018, included only as reference (green line in Fig. 3) to demonstrate the monthly alfalfa cutting cycle. The daily LAI, NDVI, and LST timeseries from MODIS and VIIRS show good temporal agreement, and are broadly consistent with variability in the Planet NDVI at USBi1.

Despite small biases seen on some days likely due to the local heterogeneity within the 500 m pixel area, both MODIS and VIIRS-based ET timeseries match well with the seasonal trends in the ET observations. The close alignment between MODIS and VIIRS-derived ET highlights the ability of VIIRS, as a follow-on instrument, in generating comparable 500-m ET estimates with MODIS and replacing MODIS as a new moderate-resolution fusion backbone. In addition, we notice the outperformance of VIIRS over MODIS in capturing the observed ET variability in a few circumstances.

For example, the underestimation of ET from MODIS for DOY 230–260 at the USBi1 site is likely due to the abnormally high MODIS LAI retrievals around DOY 240 (Fig. 3), which were physically inconsistent with the LST inputs to DisALEXI. The TSEB land-surface representation interprets elevated LST in patches of very high LAI as a signal of canopy stress, and accordingly decreases the transpiration component of ET (Kang et al. 2021). Relative to MODIS, VIIRS in general shows a better performance in ET at USBi1; even though the dip in LAI around the DOY 270 cutting (see the Planet NDVI timeseries) is not reflected in the 500-m VIIRS estimates, the retrieved LAI values remain within a reasonable range.

**Fig. 4:**
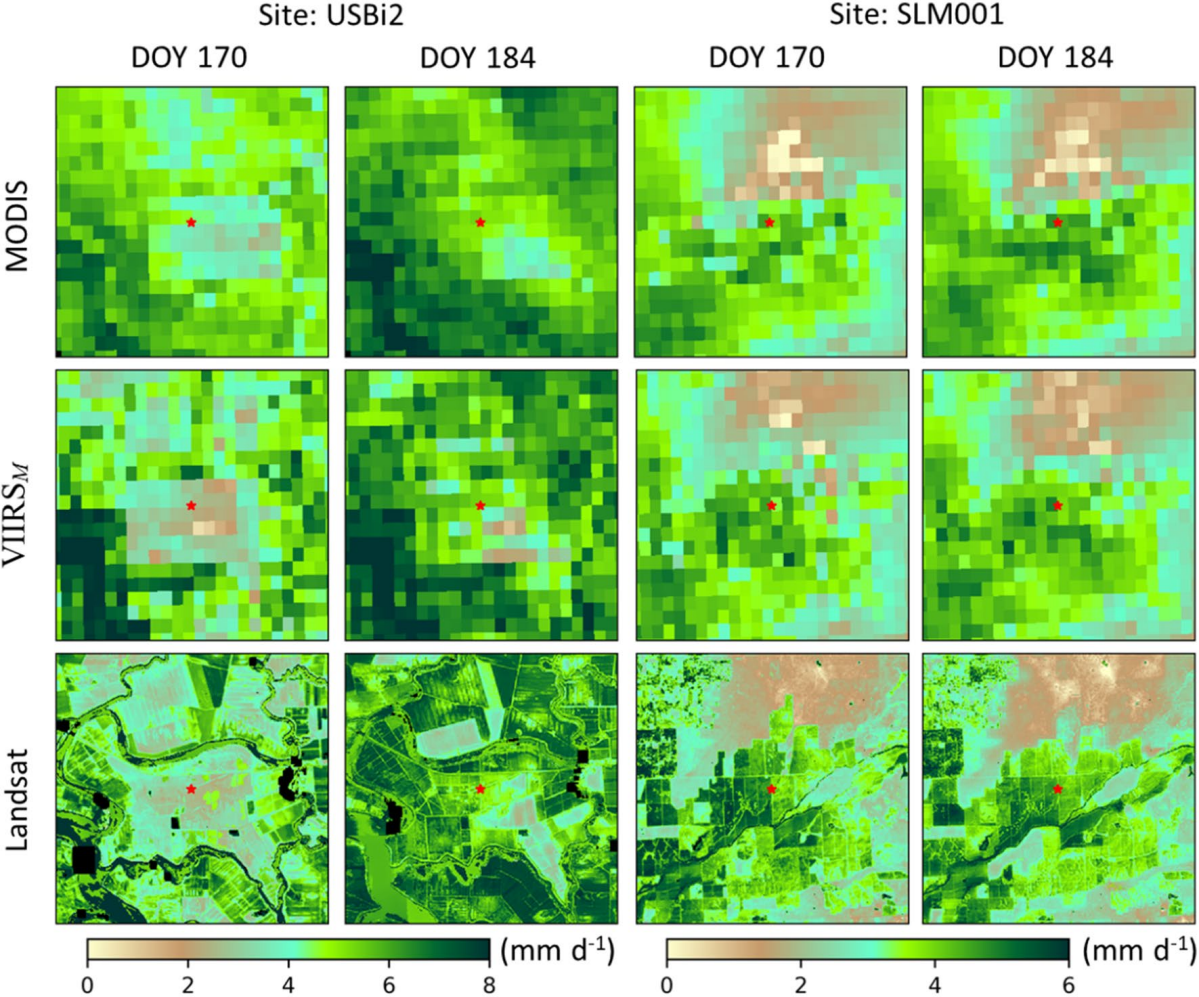
Comparison in spatial pattern of ET between MODIS (first row) and VIIRS_*M*_ (second row) on DOY 170 and 184 of 2018 over a 9 × 9 km^2^ area around towers USBi2 (left two columns) and SLM001 (right two columns). ET maps at 30-m resolution are shown in the third row for comparison. Red stars indicate the location of flux towers (colour figure online)

At the USBi2 site (corn), MODIS overestimates ET during the DOY 150–180 period before crop emergence, while VIIRS better captures this dip due to the higher values of LST retrieved during this interval (Fig. 3). Consistent with the high LST, there is no precipitation over this period. The higher native resolution of the VIIRS M thermal band (750 m) compared to MODIS (1 km) may lead to some improvement in capturing spatial details at field scale. As an example, Fig. 3 compares VIIRS_*M*_ and MODIS 500-m ET maps at USBi2 and SLM001, along with 30-m Landsat or VIIRS_*I*_/S2 ET at the same day for reference. While both MODIS and VIIRS_*M*_ reproduce the large-scale ET patterns seen in 30-m ET, the VIIRS_*M*_ ET maps exhibit a larger spatial contrast than MODIS, especially for the more heterogeneous USBi2 site. For DOY 170, the low ET values at USBi2 pre-emergence are well captured by VIIRS_*M*_ ET, but are not by MODIS. By DOY 184, the 500-m ET values are more similar near the tower (Fig. 3), although VIIRS_*M*_ shows a higher spatial variability than MODIS and is closer to the 30-m Landsat ET distribution (Fig. 4).

**Fig. 5:**
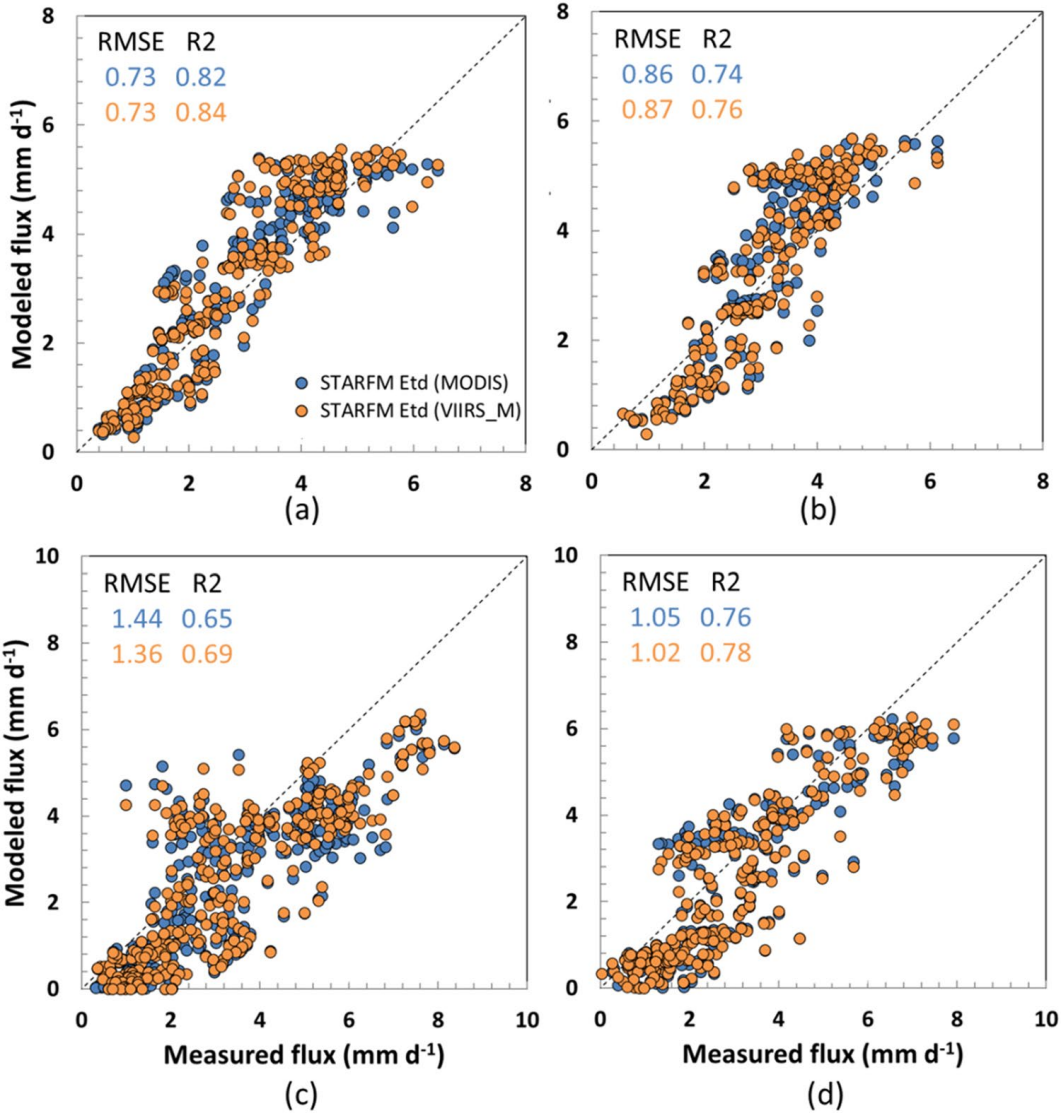
Scatter plots of fused vs. measured 30-m daily ET for year 2018 for sites **a** SLM001, **b** SLM002, **c** USBi1, and **d** USBi2. Blue dots are MODIS-based fusion results, and orange dots are VIIRS_*M*_-based fusion results, with RMSE and *R*^2^ also given in corresponding colors (colour figure online)

We further investigate the performance of DisALEXI-VIIRS_*M*_ ET as a daily moderate resolution backbone for STARM data fusion. Figure 5 shows comparisons of tower observations with daily30-m ET estimates generated by fusing MODIS and VIIRS_*M*_ 500-m ET timeseries with Landsat ET retrievals (i.e., Landsat-only fusion), respectively, over the four sites in the Lodi domain. Both MODIS and VIIRS-based fused ET show a reasonable agreement with measured fluxes, with generally improved performance from VIIRS_*M*_ with *R*^2^ ranging from 0.69 to 0.84 and RMSE ranging from 0.73 to 1.36 mm day^−1^ (Fig. 5). These results suggest that VIIRS_*M*_ will serve as a reasonable replacement for MODIS as it approaches end of life; therefore, we use VIIRS_*M*_ as the fusion backbone in the following analyses.

### Evaluation of multi-source 30-m ET timeseries

#### Evaluation on satellite overpass dates

**Fig. 6:**
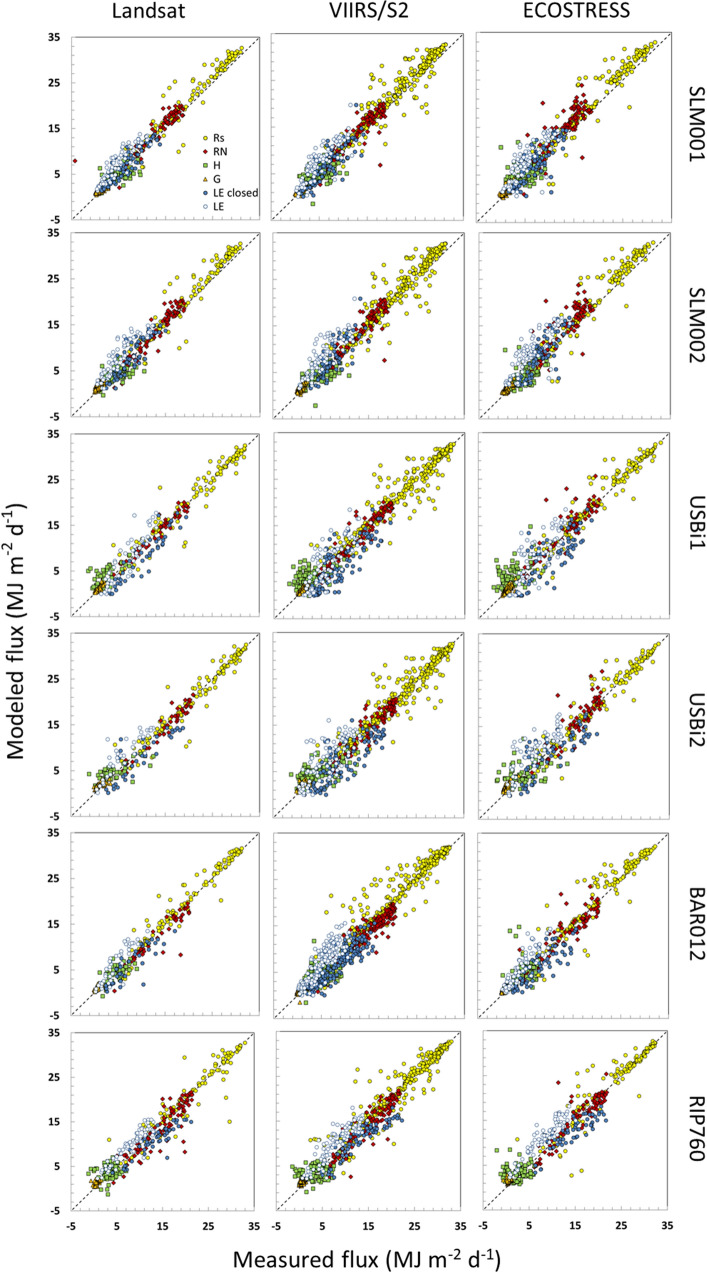
Comparisons of measured and modeled daytime integrated fluxes estimated from DisALEXI-Landsat, DisALEXI-ECOSTRESS and DisALEXI-VIIRS_*I*_/S2 on Landsat, ECOSTRESS and S2 overpass dates for years 2018–2020 at the six flux towers

In our evaluation of multi-source data fusion, we first assessed direct 30-m flux retrievals from DisALEXI-Landsat, DisALEXI-ECOSTRESS and DisALEXI-VIIRS_*I*_/S2 on satellite overpass days. Scatter plots are shown in Fig. 6 comparing modelled daytime fluxes including solar radiation, net radiation, latent heat, soil heat, and sensible heat at all six flux tower sites on Landsat, S2, and ECOSTRESS overpass dates with measurements over a 3-year period (2018–2020). For latent heat flux, the model estimates are compared to both closed and unclosed flux measurements, which collectively are assumed to bracket the ‘true’ flux. Statistical metrics of agreement between measured and modeled latent heat flux are provided in Table 3. In general, all three sets of modelled flux components agree reasonably well with measurements along the one-to-one line, suggesting a good partitioning of the surface energy budget. The DisALEXI-ECOSTRESS and DisALEXI-VIIRS_*I*_/S2 show comparable accuracy with DisALEXI-Landsat retrievals, giving similar RMSE values but providing more samples due to a higher revisit frequency. The mean RMSE of all the sites are comparable among the three data sources: approximately 1.06, 1.27, and 1.18 mm day^−1^ for DisALEXI-Landsat, DisALEXI-ECOSTRESS, and DisALEXI-VIIRS_*I*_/S2 with closed flux measurements, respectively. In most cases, model agreement is improved with the residual closure correction, except at the USBi2 site where RMSE values are relatively large.

**Table 3:** Statistical accuracy metrics for modeled daily ET flux retrievals (mm day^−1^) on Landsat, ECOSTRESS, and VIIRS_*I*_/S2 overpass dates at the six tower sites

Tower	ET	*N*	Landsat			ECOSTRESS			VIIRS_*I*_/S2		
			MAE	RMSE	MBE	MAE	RMSE	MBE	MAE	RMSE	MBE
SLM001	Unclosed	70/83/84	0.92	1.09	0.89	1.09	1.32	0.88	0.98	1.2	0.87
	Closed	69/83/83	0.56	0.83	0.14	0.73	0.95	0.04	0.61	0.82	0.04
SLM002	Unclosed	61/73/74	1.04	1.32	1	1.34	1.59	1.14	1.15	1.48	1.07
	Closed	61/73/74	0.72	0.85	− 0.04	0.89	1.08	0.15	0.84	1.06	0.02
USBi1	Unclosed	37/56/72	0.97	1.23	0.3	1.16	1.48	0.47	1.02	1.26	0.2
	Closed	43/69/97	0.95	1.13	− 0.66	1.24	1.46	− 0.55	1.18	1.4	− 0.69
USBi2	Unclosed	34/58/78	1.11	1.42	0.2	1.50	1.86	0.56	1.35	1.66	0.25
	Closed	36/64/95	1.34	1.75	− 0.86	1.12	1.94	− 0.55	1.49	1.81	− 0.76
BAR012	Unclosed	40/70/168	0.69	0.9	0.46	0.77	1.04	0.61	0.79	1.05	0.58
	Closed	39/70/167	0.58	0.82	− 0.38	0.69	0.9	− 0.35	0.67	0.83	− 0.39
RIP760	Unclosed	65/69/89	0.64	0.78	0.49	1.02	1.21	1	0.8	1.02	0.68
	Closed	51/56/74	0.81	0.99	− 0.63	0.83	1.02	− 0.34	0.8	0.96	− 0.47
All sites	Unclosed	307/409/565	0.88	1.12	0.62	1.13	1.43	0.8	0.97	1.25	0.61
	Closed	299/415/590	0.79	1.06	− 0.33	0.97	1.27	− 0.25	0.92	1.18	− 0.4

**Fig. 7:**
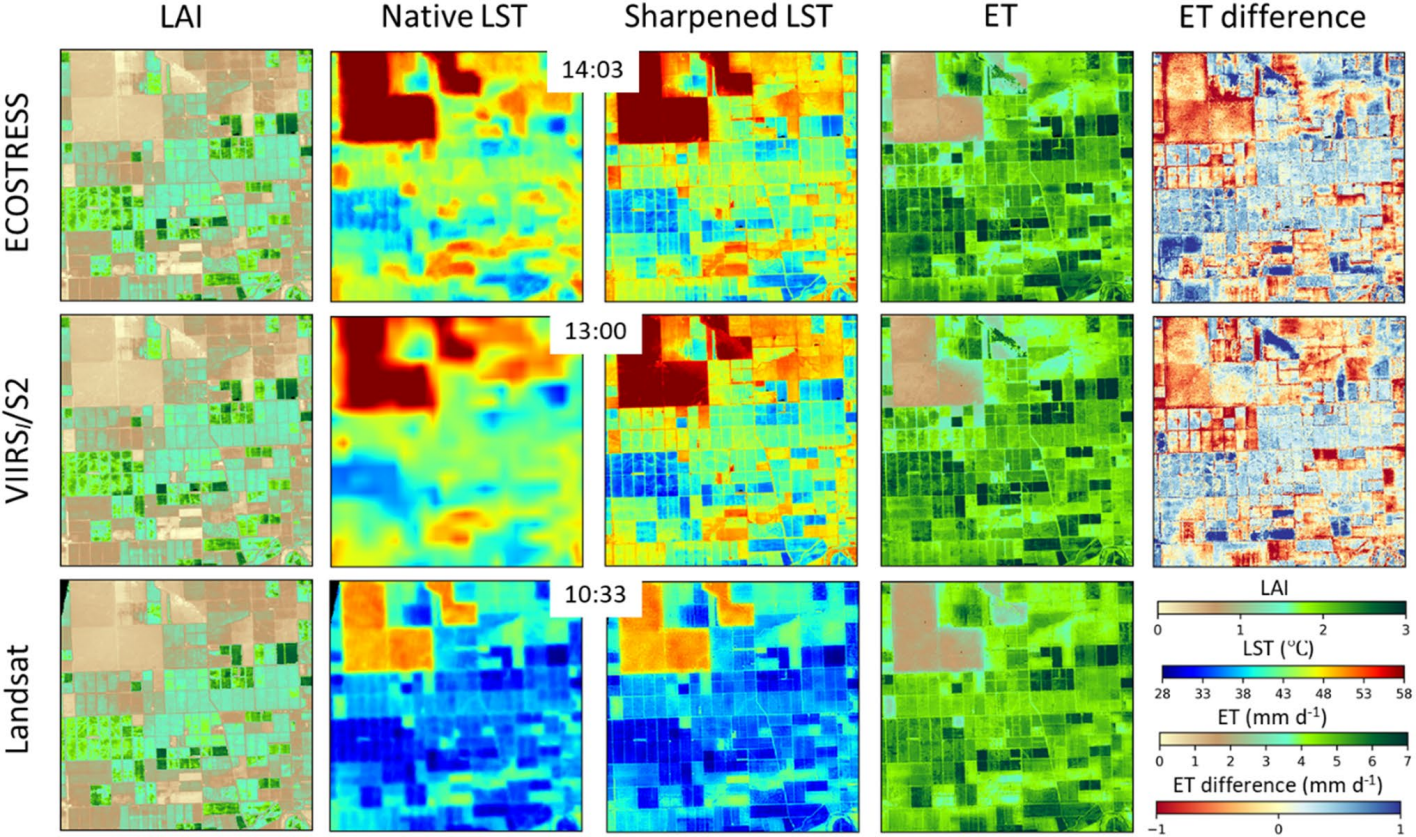
Spatial maps of LAI (first column), LST at native resolution (°C; second column), sharpened LST (°C; third column), and daily ET retrievals (mm day^−1^; four column) for DisALEXI-ECOSTRESS (top row; 2018217), DisALEXI-VIIRS_*I*_/S2 (middle row; 2018217), and DisALEXI-Landsat (bottom row; 2018218) over a subset of the Madera domain. ET difference maps (ECOSTRESS (VIIRS_*I*_/S2) minus Landsat) are shown in top (middle) panel of the fifth column, respectively. The acquisition time of LST data is also listed

In addition to point comparison of time-series at tower sites, the agreement in spatial pattern among the three sets of ET retrievals and key inputs was also assessed. Figure 7 shows an example comparison over a sub-region in the Madera domain in 2018. Here, the ECOSTRESS and VIIRS_*I*_/S2 maps are from DOY 217, while the Landsat maps are from DOY 218 as the same-day overpass is unavailable. However, there was no precipitation on either day and the mean air temperature and insolation rates measured at the tower were similar, allowing a reasonably fair comparison. In general, the ET patterns are similar for the three thermal data sources. The LAI data used for both DisALEXI-ECOSTRESS and DisALEXI-VIIRS_*I*_/S2 modeling are from S2 on DOY 217, and show good agreement with Landsat LAI from DOY 218. The VIIRS LST map has less spatial contrast due to the much coarser native TIR resolution (Bellvert et al. 2020; Xue et al. 2021); however, the DMS sharpening approach effectively improves the spatial contrast and detail, resulting in comparable spatial structure between the three sensors. The remaining differences in LST are largely due to the different times of acquisition, with progressively hotter temperatures as we move through the diurnal cycle. Despite these temporal differences in LST, the spatial patterns of daily ET from the three sources are consistent due to the multi-scale constraints inherent in the ALEXI/DisALEXI modeling system.

#### Evaluation of fused daily timeseries

**Fig. 8:**
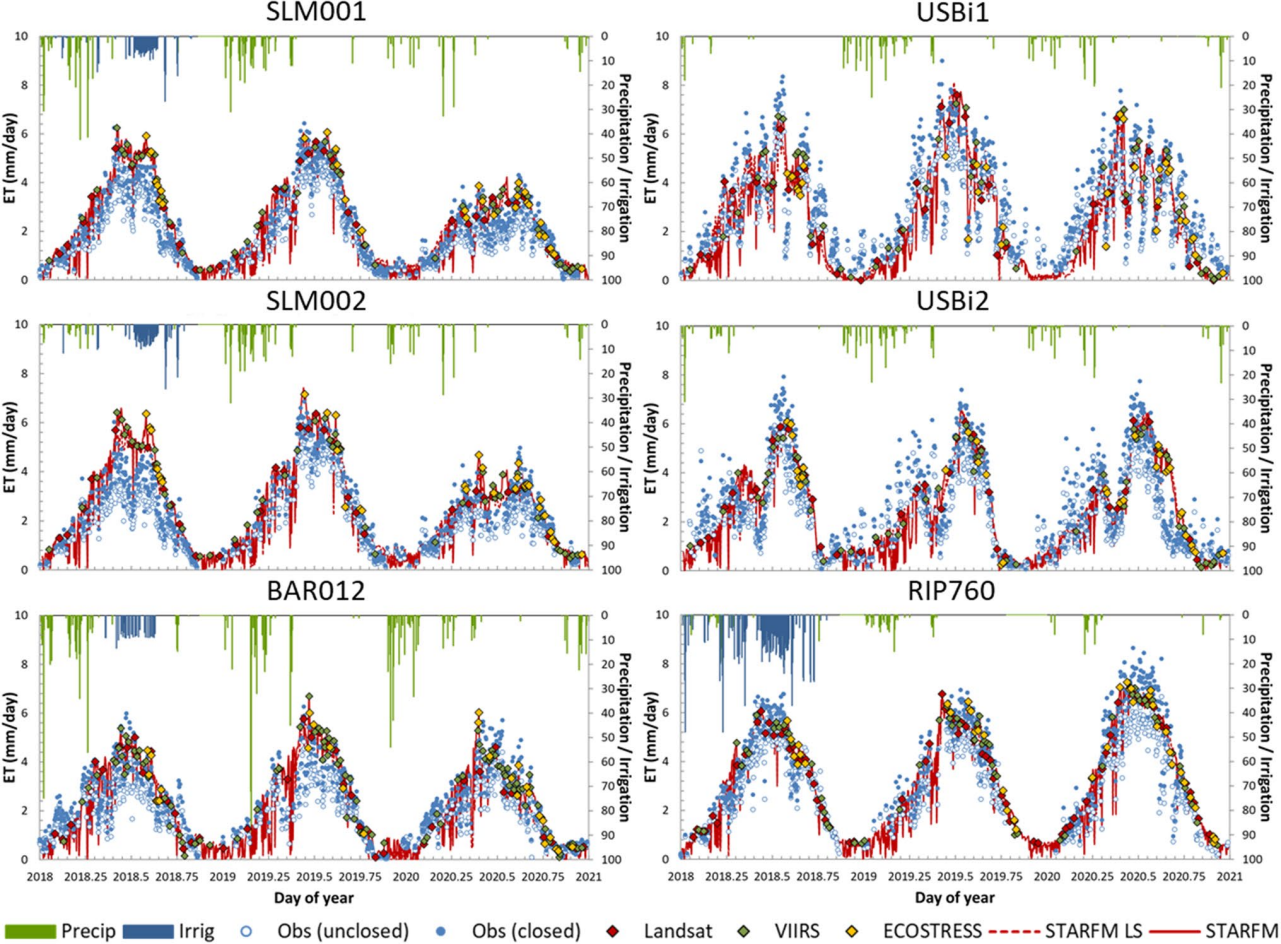
Time series comparison between measured and modeled daily ET obtained from both Landsat-only and Landsat + ECOSTRESS + VIIRS_*I*_/S2 fusion at the six flux tower sites for the 3-year study period

The comparable quality of the three ET datasets on satellite overpass dates suggests utility for combined use as layers within the ET fusion method, generating 30-m daily ET timeseries. Two ET datacubes were constructed by fusing 500-m VIIRS_*M*_ ET with Landsat (Landsat-only fusion) and with Landsat, ECOSTRESS and VIIRS_*I*_/S2 (Landsat + ECOSTRESS + VIIRS_*I*_/S2 fusion) over the period of 2018–2020. Time series of fused daily ET from both datacubes are compared with flux tower observations in Fig. 8. The modelled ET on Landsat, ECOSTRESS and S2 overpass dates, and daily total of rainfall and irrigation applied are also indicated in Fig. 8. Overall, both fusion schemes well capture the seasonal dynamics in ET measurements at all six sites. At SLM001 and SLM002, the Pinot noir vines were cut and grafted to Cabernet Sauvignon and Merlot, respectively, at the beginning of 2020, gradually regrowing through the course of the season. The relatively lower ET in 2020 than 2018 and 2019 and the regrowth trends in 2020 were reasonably diagnosed by the model with no a priori information on management.

Statistical metrics in terms of MAE, RMSE, MBE and RE between modelled and observed ET at daily and weekly scales for both fusion schemes are given in Table 4. Both fusion schemes give MAE (RMSE) of 0.52–0.69 (0.69–0.94) mm day^−1^ at a daily timestep for all four vineyard flux sites. The results are consistent with the previous ALEXI/DisALEXI-based studies at vineyard sites with RMSE ranging between 0.7 and 1.0 mm day^−1^ (Anderson et al. 2018; Knipper et al. 2020, 2019a; Semmens et al. 2016; Xue et al. 2021) and other remote sensing-based models such as METRIC with RMSE of 0.6–1.1 mm day^−1^ (Carrasco-Benavides et al. 2012; Galleguillos et al. 2011). This also aligns with the target model error of ± 0.8 mm day^−1^ suggested by Seguin et al. (1999) for the field scale of agricultural and hydrological studies. Irrigation decision making often occurs at the weekly timescale, where MAE (RMSE) reduces to 0.44–0.62 (0.56–0.87) mm day^−1^ due to time averaging of random errors. The model errors are more pronounced at the USBi1 and USBi2 sites relative to the vineyard sites, in part due to the inherent greater ET temporal variation at these sites (Xue et al. 2021).

**Table 4:** Statistical metrics of comparison between measured (closed) and modeled ET (mm day^−1^) obtained from both Landsat-only and Landsat + ECOSTRESS + VIIRS_*I*_/S2 fusion at daily and weekly timesteps over the 3-year study period

Domain	Tower	Timescale	*N*	Landsat-only				Landsat + ECO + VIIRS_*I*_/S2			
				MAE	RMSE	MBE	%RE	MAE	RMSE	MBE	%RE
Lodi	SLM001	Daily	722	0.52	0.69	0.24	20.6	0.55	0.75	0.29	21.8
		Weekly	90	0.44	0.56	0.26	16.6	0.49	0.63	0.32	18.2
	SLM002	Daily	618	0.60	0.80	0.26	22.1	0.69	0.94	0.41	25.1
		Weekly	76	0.54	0.72	0.26	18.6	0.62	0.87	0.43	21.1
	USBi1	Daily	1044	1.19	1.42	− 0.77	37.5	1.08	1.29	− 0.69	34.2
		Weekly	146	1.07	1.21	− 0.75	33.3	0.95	1.07	− 0.67	29.8
	USBi2	Daily	985	1.27	1.64	− 0.73	40.8	1.27	1.62	− 0.77	40.8
		Weekly	136	1.18	1.50	− 0.74	37.0	1.18	1.47	− 0.78	37.1
Cloverdale	BAR012	Daily	653	0.59	0.78	0	24.1	0.57	0.75	0.06	23.6
		Weekly	76	0.51	0.63	0	20.0	0.47	0.57	0.06	18.3
Madera	RIP760	Daily	521	0.67	0.87	− 0.20	14.7	0.67	0.87	− 0.10	14.8
		Weekly	67	0.50	0.63	− 0.16	10.5	0.49	0.63	− 0.05	10.2
All	All sites	Daily	4543	0.87	1.18	− 0.28	28.7	0.86	1.15	− 0.23	28.4
		Weekly	591	0.79	1.05	− 0.30	25.0	0.78	1.01	− 0.24	24.4

At all but the SLM vineyard sites, performance in daily and weekly ET improved in terms of MAE, RMSE and/or MBE with the extra sampling in the Landsat + ECOSTRESS + VIIRS_*I*_/S2 fusion, as did the Nash–Sutcliffe coefficient of efficiency (not shown). At the SLM sites, performance was slightly disimproved with the inclusion of ECOSTRESS and VIIRS_*I*_/S2 in these retrospective analyses. At these sites, the temporal dynamics were already reasonably reproduced using Landsat alone, and adding the lower quality supplementary retrievals (especially ECOSTRESS, see Table 3) served to add noise to the timeseries. In future studies, a weighting scheme will be evaluated, enabling differential strength of contribution to the fused timeseries based on sensor retrieval accuracy (Table 3). We note, however, that the multi-source retrievals do add value at these sites in real-time mode (see “Value for real-time applications”).

### Added value of extra temporal sampling

#### Capturing rapid changes

**Fig. 9:**
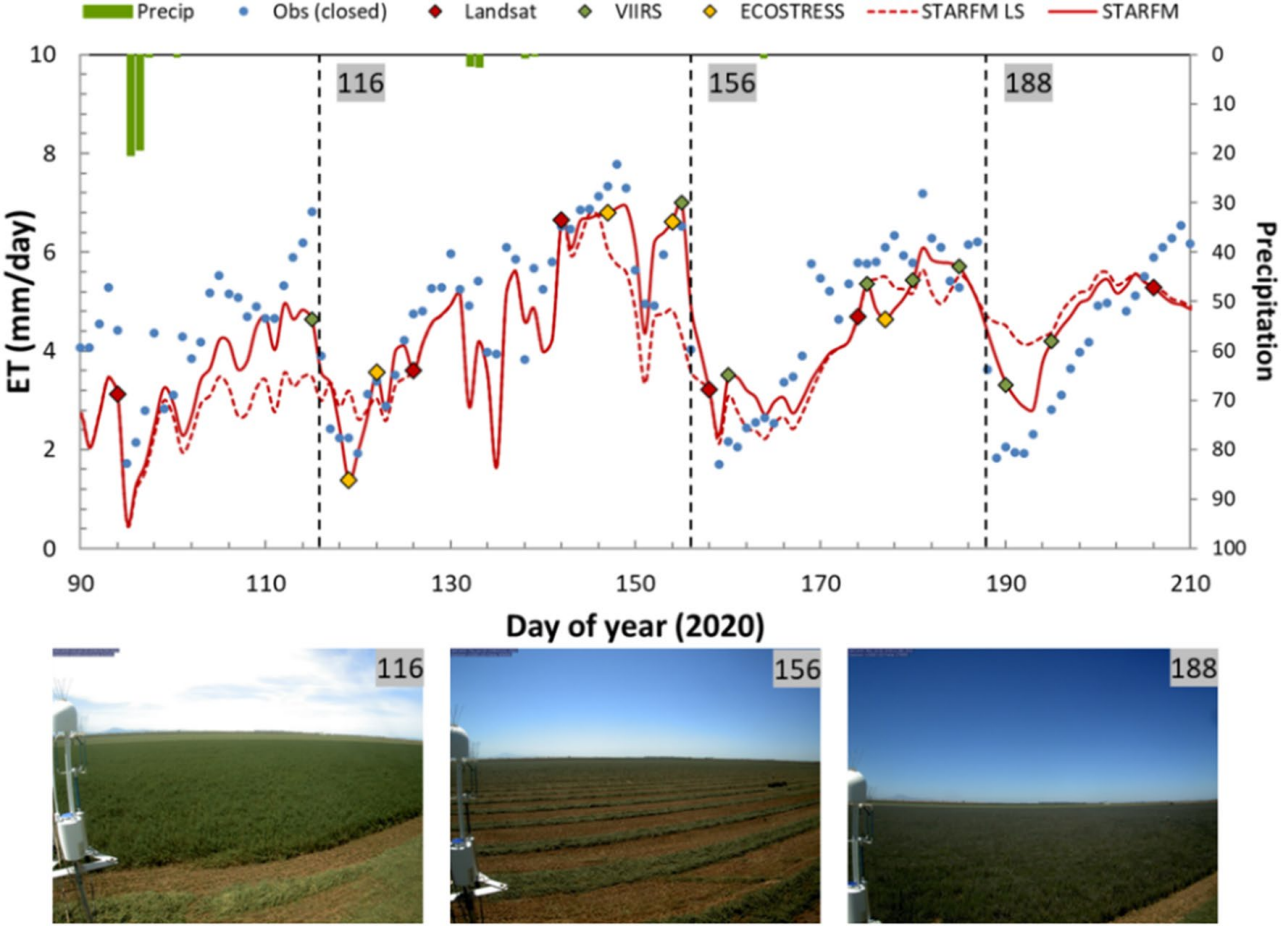
Model-measurement timeseries comparison of daily ET at the USBi1 site during DOY 90–210, 2020 (upper panel). The dashed vertical lines indicate the alfalfa cutting dates. Bottom panel, photographs from a PhenoCam at the USBi1 site showing alfalfa cuttings on dates highlighted in the upper timeseries plot

At many sites, the Landsat + ECOSTRESS + VIIRS_*I*_/S2 fusion shows an improved performance in capturing ET temporal dynamics than Landsat-only fusion, yielding smaller errors in comparison with observations. For instance, at the USBi1 site the incorporation of ECOSTRESS and VIIRS_*I*_/S2 data results in a reduction in RMSE from 1.42 to 1.29 mm day^−1^ and 1.21 to 1.07 mm day^−1^ at daily and weekly time steps (Table 4), respectively. The monthly cutting of alfalfa and following quick regrowing result in strong ET temporal variations, making it difficult for Landsat-only fusion to reproduce the frequent ET changes due to insufficient Landsat scenes. The extra ECOSTRESS and VIIRS_*I*_/S2 samples provide key complements to Landsat in capturing the monthly peaks and lows. Figure 9 shows one representative example demonstrating the significant benefit of extra samples in helping capture ET dynamics. For the period of DOY 90–210 in 2020, the alfalfa at USBi1 was cut three times on DOY 116, 156 and 188, respectively. The ECOSTRESS retrieval on DOY 119 and VIIRS_*I*_/S2 retrieval on DOY 190 lead to improved estimates of the low ET after cutting in comparison with Landsat-only fusion. During DOY 100–115, the increasing trend of ET as the crop with increasing alfalfa biomass is not captured by Landsat-only fusion. The VIIRS_*I*_/S2 ET estimate on DOY 115 elevates the fused ET timeseries to be closer to the observations.

**Fig. 10:**
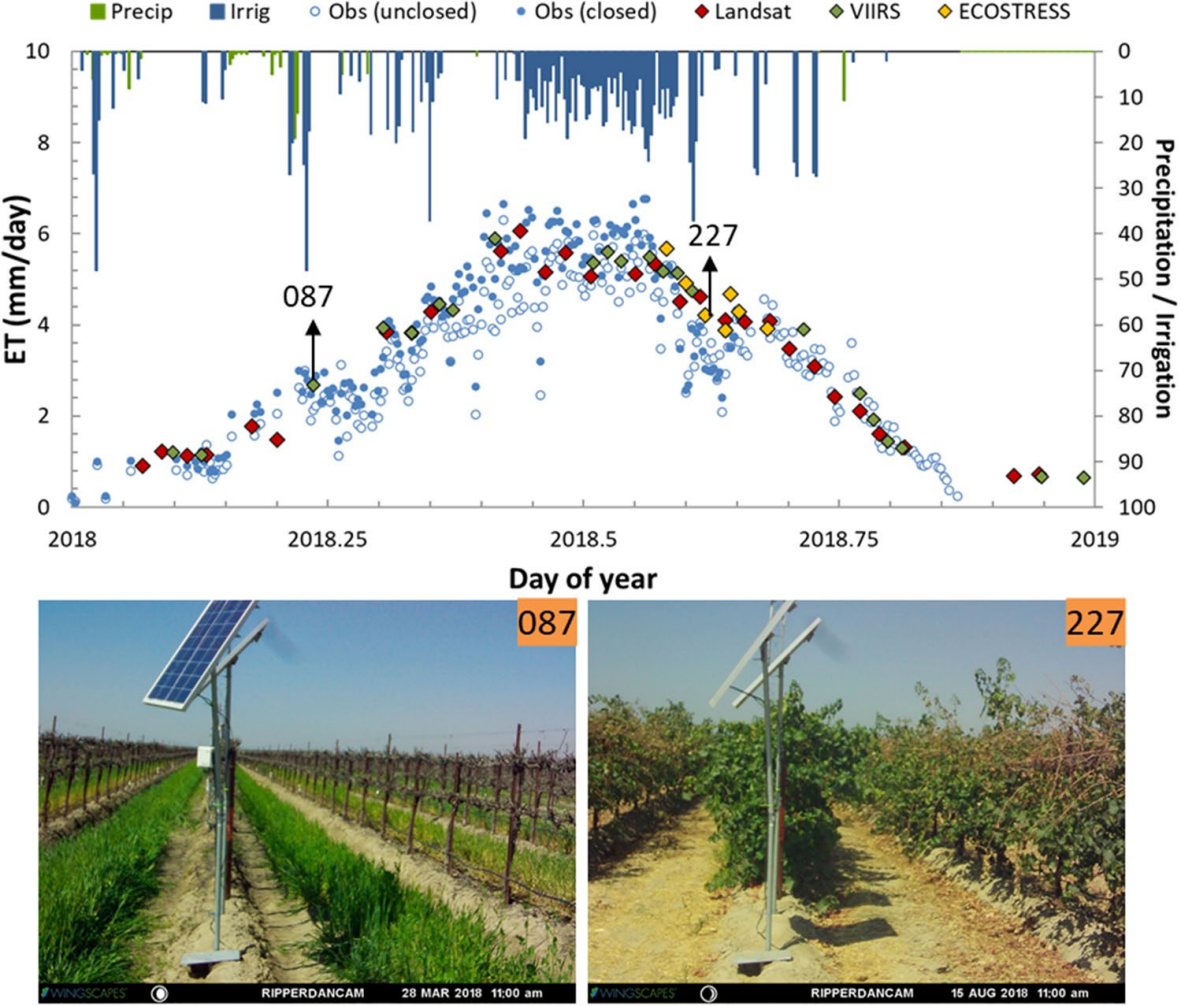
Comparison between measured and modeled ET retrievals obtained from DisALEXI-Landsat, DisALEXI-ECOSTRESS, and DisALEXI-VIIRS_*I*_/S2 at RIP760 in 2018 (top panel). Photographs in the bottom row are from a PhenoCam in the RIP760 site and show vineyard canopy conditions on dates highlighted in the top panel

Additional ECOSTRESS and VIIRS_*I*_/S2 samples also improved ET estimates at the BAR012 site, reducing relative errors from 20 to 18% at the weekly timescale (Table 4). Further to the south in California, where clear-sky conditions are more prevalent, extra sampling from ECOSTRESS and VIIRS_*I*_/S2 did not lead to significant improvement in the overall statistical metrics at SLM and RIP760 site. Still, these extra samples are important in better defining local water use at key phenological stages during growing season. Looking in greater detail at the multi-source ET retrievals in 2018 over RIP760 site in Fig. 10, we can see the added value of extra sampling. For example, a few ECOSTRESS samples over DOY 210–240 facilitated a better definition of the reduction in ET timeseries during the veraison to post-veraison stage from late July or early August (Kustas et al. 2018) in which the irrigation was curtailed (Fig. 10). During this period, the vines are still actively transpiring with the ripening of the fruit until the grapes reach the required sugar content by late August or early September for harvesting. In addition, one additional VIIRS_*I*_/S2 sample on DOY 87 effectively filled a large gap in springtime (DOY 75–115), during which there are no clear-sky Landsat overpasses. This period of vine phenological stages from bud break to flowering with low vine cover typically has significant cover crop biomass (Fig. 10). An ET acquisition near the bud break (around DOY 90)—start of the vine growing season- is very important for monitoring cover crop water use and in making decisions on initiating irrigation.

#### Value for real-time applications

**Fig. 11:**
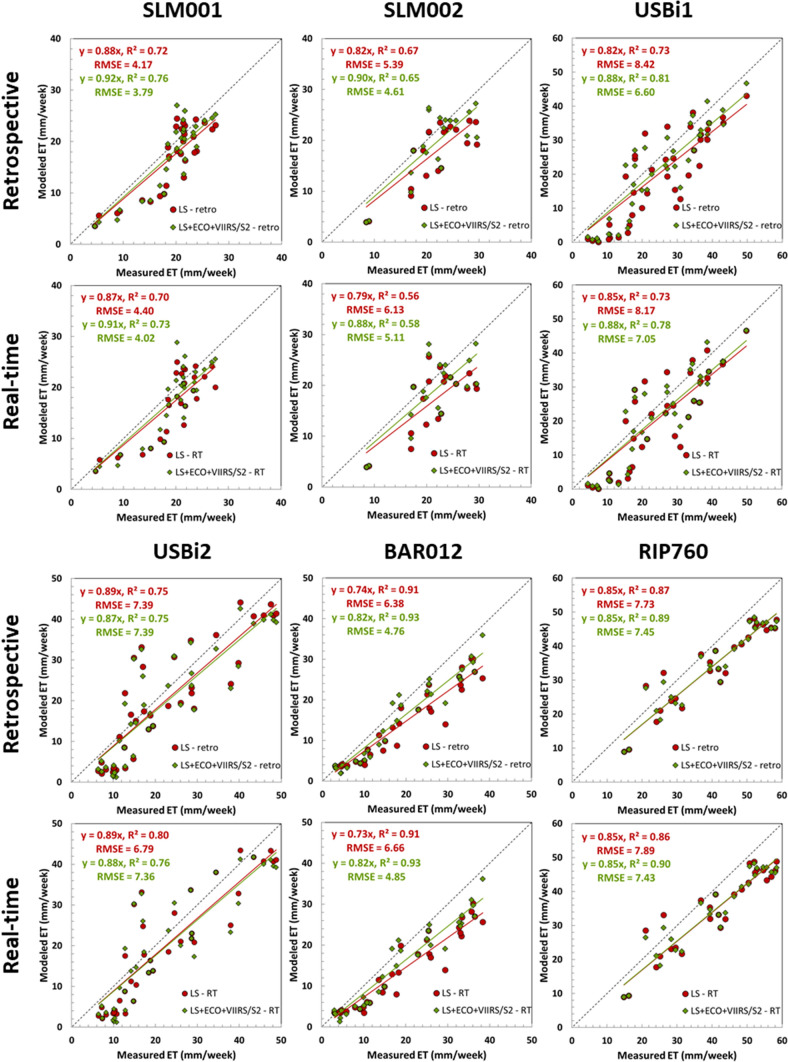
Scatter plots of measured and modeled weekly ET in 2020 from both Landsat-only (LS) and Landsat + ECOSTRESS + VIIRS_*I*_/S2 (LS + ECO + VIIRS/S2) fusion for both retrospective (-retro) and real-time (-RT) modeling schemes over the six flux tower sites. The red and green lines indicate linear regression for LS and LS + ECO + VIIRS/S2 fusion, respectively. The intercept of the regression is forced to zero

The ET fusion results in “Evaluation of fused daily timeseries” and “Capturing rapid changes” were generated retrospectively by using all image-pairs available before and after each prediction date. In this case, clear-sky Landsat overpasses may often be sufficient to capture the ET dynamics over vineyard sites in central and southern California. However, for operational applications, only the image-pair before the prediction date will be available, which tends to exacerbate the disadvantages of insufficient Landsat overpasses and highlight the value of extra temporal samplings from ECOSTRESS and VIIRS_*I*_/S2. The importance of temporal sampling frequency for real-time applications was examined in this study. While data latency was not considered in this assessment, it should be noted that the added value will become more pronounced if there is a significant delay between data acquisition and public availability. We compared the weekly total ET from retrospective and real-time fusion modes for both Landsat-only and Landsat + ECOSTRESS + VIIRS_*I*_/S2 fusion with measured ET at the six sites for year 2020 (Fig. 11). For both Landsat-only and Landsat + ECOSTRESS + VIIRS_*I*_/S2 fusion approaches, the retrospective case exhibits a better performance than the real-time case for most of sites. In addition, for both real-time and retrospective applications the Landsat + ECOSTRESS + VIIRS_*I*_/S2 outperforms the Landsat-only fusion for all sites except USBi2—the regression line is closer to the one-to-one line with a lower RMSE. For all four vineyard sites (SLM001, SLM002, BAR012, and RIP760), the Landsat + ECOSTRESS + VIIRS_*I*_/S2 fusion outperforms the Landsat-only fusion, reducing RMSE by 9.1%, 14.5%, 25.4% and 3.6% for the retrospective case and by 8.6%, 16.6%, 27.2% and 5.8% for real-time application, respectively.

**Fig. 12:**
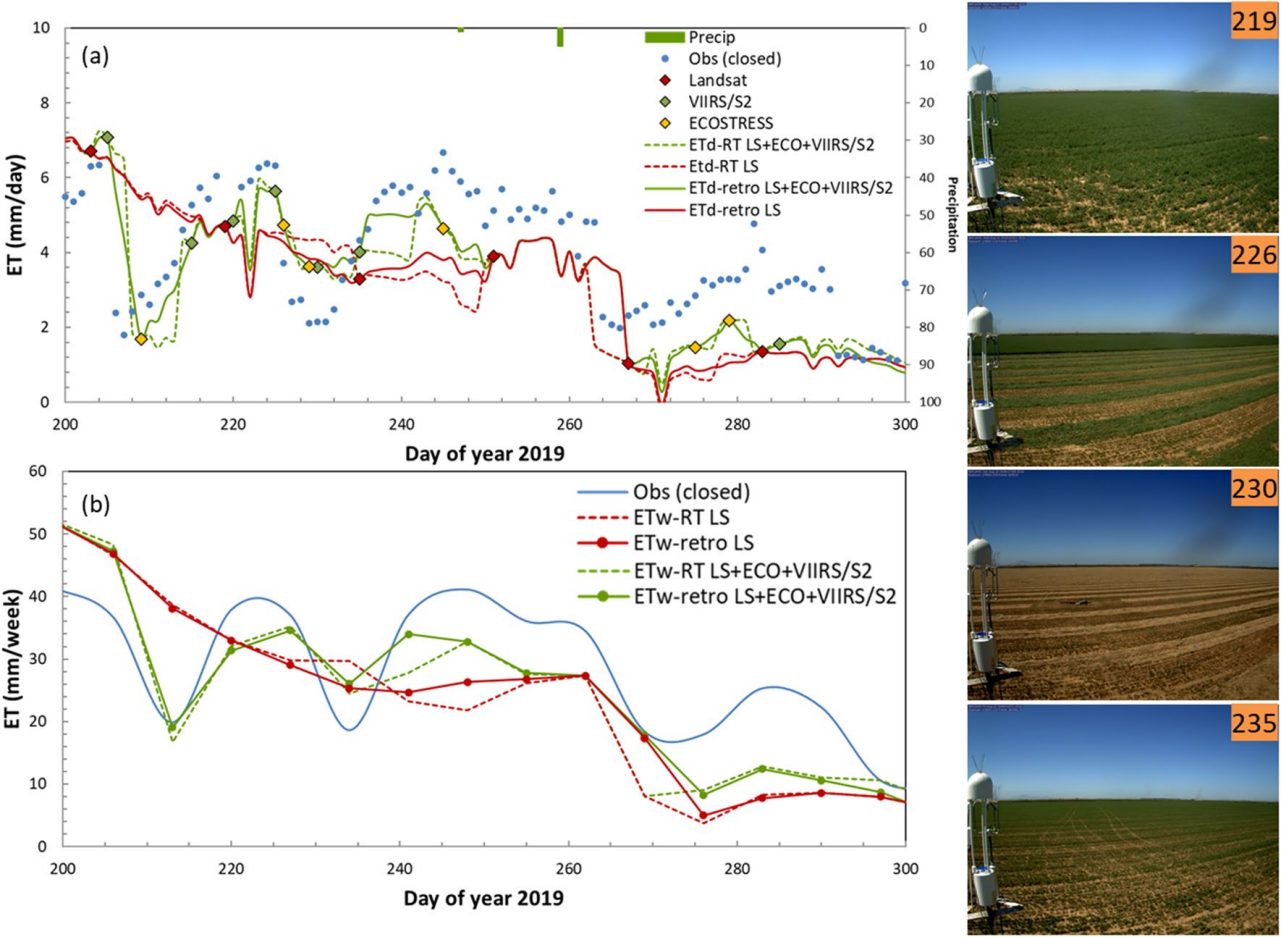
Daily (upper-left panel) and weekly total (bottom-left panel) ET timeseries in both retrospective and operational modeling schemes at the USBi1 tower site during DOY 200–300 in 2019. Photographs (right panel) from a PhenoCam at USBi1 site showing the temporal evolution of alfalfa canopy biomass on some key days

In addition to these overall statistics, we further use USBi1 site in 2019 to demonstrate the increased value of extra sampling in improving real-time ET estimates at both daily and weekly time steps (Fig. 12). Overall, the Landsat + ECOSTRESS + VIIRS_*I*_/S2 fusion exhibits improved skill relative to Landsat-only fusion in capturing the temporal dynamics of measured ET for both retrospective and real-time schemes (Fig. 12). During DOY 220–280, the Landsat-only fusion gives somewhat different assessments of ET temporal dynamics in retrospective and real-time mode, while timeseries using Landsat + ECOSTRESS + VIIRS_*I*_/S2 fusion are more similar, particularly at weekly timestep. For example, for the week ending on DOY 234 (DOY 228–234), Landsat-only fusion in real-time mode cannot project the reduction in ET during the cutting cycle because it is using only a pre-cutting retrieval from DOY 219 (see PhenoCam photos in Fig. 12). This reduction is better captured in real-time with the multi-source timeseries. Similarly, the regrowth pattern for weeks ending on DOY 241 and 248 is poorly reconstructed in real-time with Landsat alone, but is better sampled with Landsat + ECOSTRESS + VIIRS_*I*_/S2 fusion in both modes.

### Detection of vineyard water stress and changes in management

**Fig. 13:**
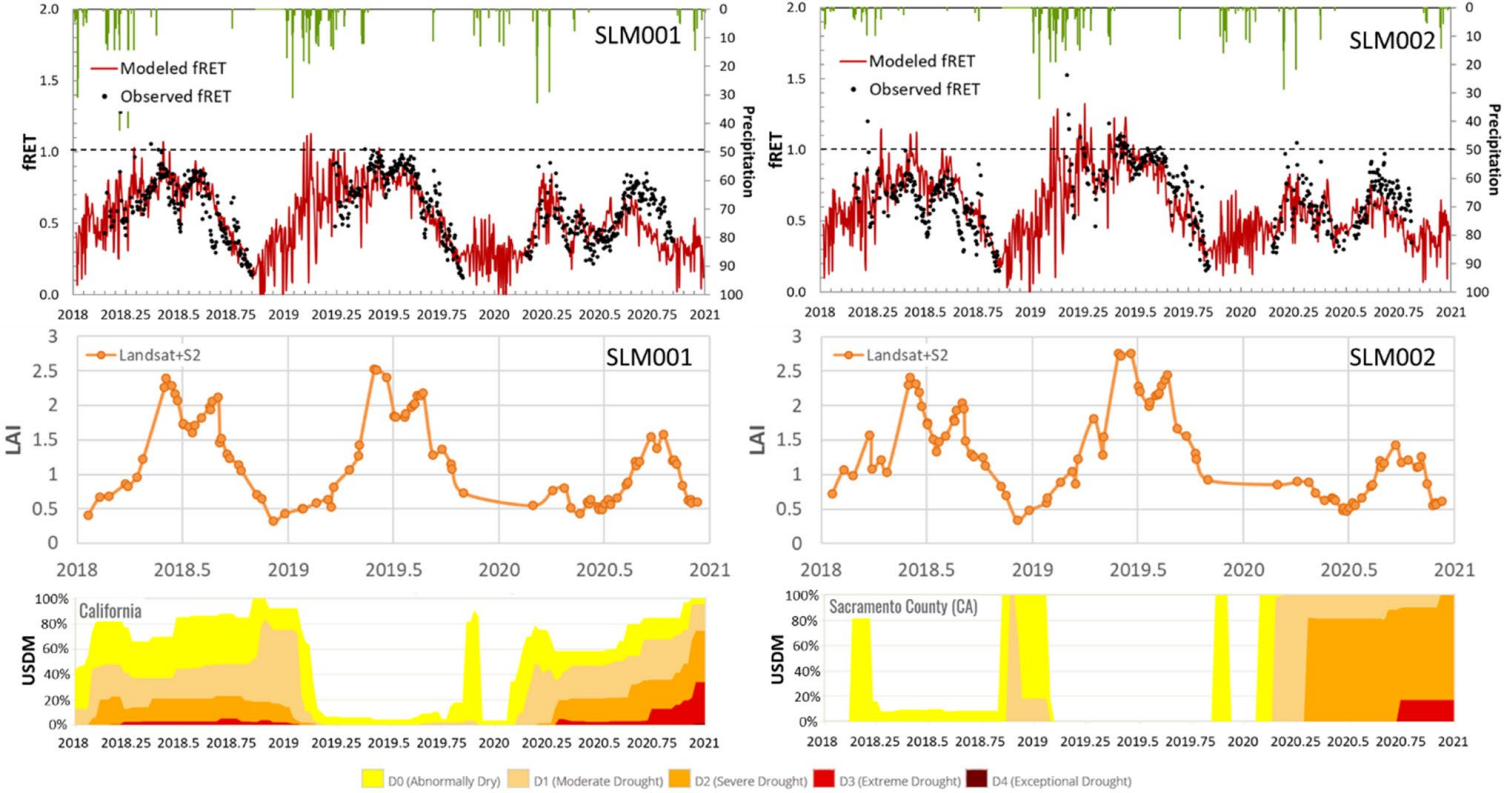
Timeseries of 30-m daily *f*_RET_ obtained from both flux tower observations (black dots) and fused ET datacube (red line) over SLM001 (top left panel) and SLM002 site (top right panel) during 2018–2020, along with daily rainfall (mm; green bars). LAI timeseries on both Landsat and S2 overpass dates for the two sites (middle row). Timeseries of percent area in USDM categories for California state (bottom left panel) and Sacramento County (bottom right panel), respectively (colour figure online)

The growth, quality and productivity of vine grapes largely depend on the water status. Regulated deficit irrigation (RDI) is one of the strategies used to optimize the quality of vines by imposing some degree of water stress in the vineyards during specific growing periods (McCarthy et al. 2002). The moisture stress factor *f*_RET_ describing the ratio of actual to reference ET is related to the crop management factor (defined as the ratio of actual water use to crop water requirement) often used in RDI to track the spatial and seasonal patterns of vineyard water status in real time. Figure 13 shows a comparison between modeled and measured daily 30-m *f*_RET_ timeseries over the SLM001 and SLM002 sites. The modeled actual ET is extracted from the Landsat + ECOSTRESS + VIIRS_*I*_/S2 fusion results and the measured actual ET is computed from tower observations with closure. The modeled reference ET is calculated using regional meteorological data while the measured reference ET is computed using flux tower measured data. Also shown are the remote sensing LAI timeseries on both Landsat and S2 overpass dates over the two sites (middle row), indicating the phenological development of vine grapes. The USDM timeseries over the state of California and over Sacramento County where the two sites are located are also shown in Fig. 13 (bottom row), reflecting ambient moisture conditions.

For the two vineyard sites (SLM001 and SLM002), the modeled *f*_RET_ time series from Landsat + ECOSTRESS + VIIRS_*I*_/S2 fusion agree well with the tower measurements (Fig. 12), suggesting that remote sensing can effectively supplement in situ observations used to track stress and inform vineyard irrigation. We note some negative bias in modeled *f*_RET_ in the second half of 2020 as the vines in both blocks start to regrow after grafting (see the LAI timeseries). Lower actual ET observed in these blocks in 2020 is due in large part to reduced biomass post-grafting, but may have been exacerbated by severe ambient drought conditions as reported in the USDM for Sacramento County (Fig. 13). The influence of both management practices and drought conditions is reflected in the modeled *f*_RET_ during the growing season, approximately 0.6–0.7 for 2020 and 0.8–1.0 (close to target level) for 2018–2019.

**Fig. 14:**
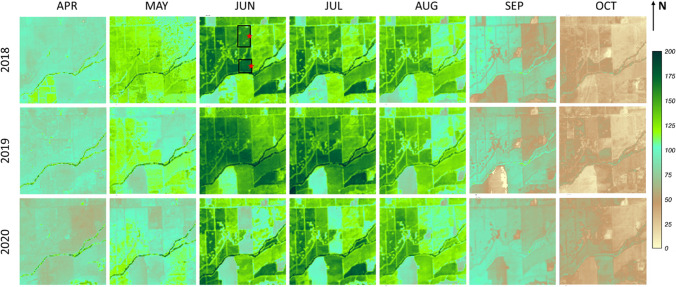
Spatial maps of monthly ET (mm/month) over a 3 × 3 km^2^ area surrounding the SLM001 and SLM002 vineyards for the growing season during 2018–2020. Black boxes indicate the two vineyard blocks (north: SLM001; south: SLM002). The red stars indicate the locations of flux tower sites

**Fig. 15:**
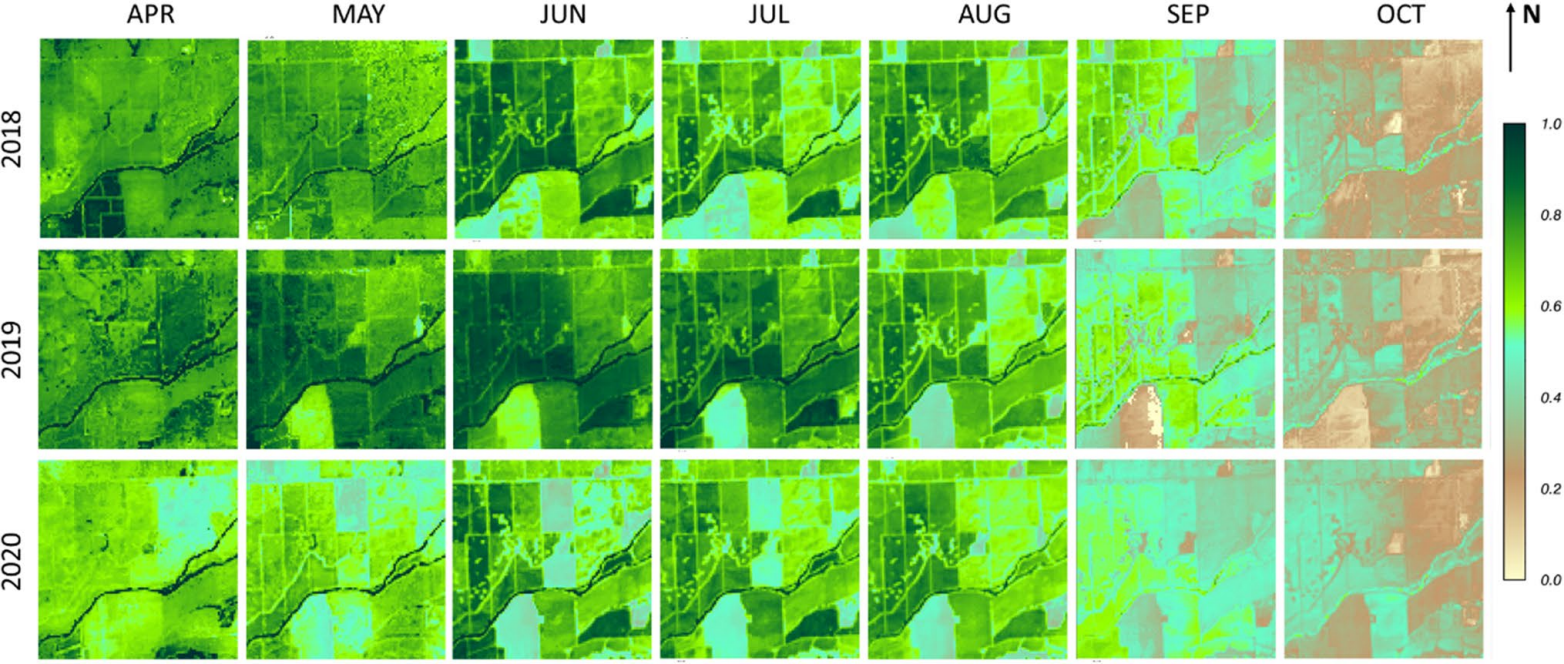
Spatial maps of monthly *f*_RET_ over a 3 × 3 km^2^ area surrounding the SLM001 and SLM002 vineyards for the growing season during 2018–2020

**Fig. 16:**
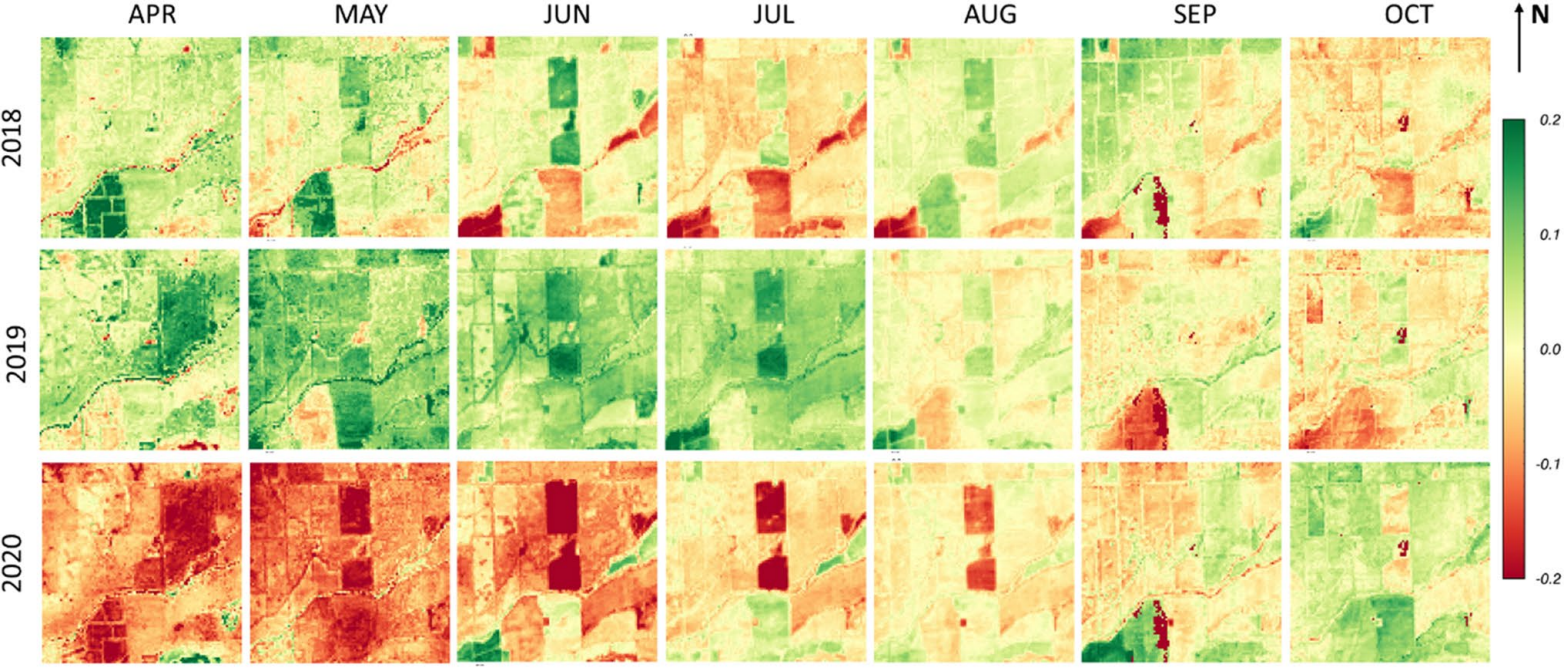
Spatial maps of monthly *f*_RET_ anomaly (Δ*f*_RET_) over a 3 × 3 km^2^ area surrounding the SLM001 and SLM002 vineyards for the growing season during 2018–2020

Maps of water use and stress over this three-year interval (Figs. 14, 15, 16) give spatial context for the temporal behavior described in Fig. 13. Figure 14 shows the monthly ET over a 3 × 3 km^2^ area surrounding the SLM001 and SLM002 vineyard site. The seasonal trends in water use are relatively consistent between 2018 and 2019: ET increases in the irrigated vineyards starting in the bud break and flowering stages in May, reaches peak values in June and July when the vines and fruit are growing rapidly, declines gradually after August in the veraison and post-veraison stage, then drops rapidly after harvest in early September with very little evaporation occurring in October (Kustas et al. 2018). In 2020, most blocks show a similar pattern to 2018–2019 but relatively lower ET values perhaps related to the drought. The reduced ET in the SLM blocks due to the grafting is readily apparent in 2020.

In comparison with actual ET, *f*_RET_ is a better indicator of water stress since it reduces the dependence on seasonal atmospheric variations and radiation load and more focuses on variations in surface moisture conditions and plant status. Spatiotemporal patterns of monthly *f*_RET_ at 30-m resolution (Fig. 15) also reflect the impact of management practices. In contrast to 2018–2019, for 2020 *f*_RET_ in the two vineyard blocks remains below approximately 0.5 before August, reflecting the lower biomass and transpiration flux after grafting. Higher overall levels of *f*_RET_ in 2019 in April–July compared to 2018 and 2020 may be related to drought relief in that year.

These temporal differences in moisture and plant status are highlighted in monthly *f*_RET_ anomaly (Δ*f*_RET_) maps computed relative to the 3-year monthly means (Fig. 16). From April to August in 2020, the reduced water use in the grafted SLM blocks is clearly indicated by significant negative anomalies. Negative anomalies were also found in surrounding fields in 2020, indicating water stress likely related to the regional drought conditions. In addition to crop management practices, Δ*f*_RET_ also represents changes associated with land cover/ land use. For example, the negative anomalies in the alfalfa field south of the SLM002 vineyard in June and July 2018 change to positive in 2019 when a major land cover change is from alfalfa to winter wheat. Drier than normal conditions in the south-west corner in most period of 2018 growing season also becomes positive in 2019–2020 as the dominate crop type changes from winter wheat to alfalfa.

In summary, spatial maps of monthly ET, *f*_RET_ and Δ*f*_RET_ at 30-m scale can help to monitor changes in local and regional water use, crop management practices and land use, providing valuable spatial and temporal information for decision makers to assess conditions and develop short- and long-term management strategies.

## Discussion

### Limitations and opportunities in multi-source ET mapping

ET estimates with high resolution both spatially and temporally have demonstrated value for operational agricultural water management, including guiding precision irrigation at sub-field scale (Knipper et al. 2019b). In “Results”, we have demonstrated the added value of extra temporal samples from ECOSTRESS and VIIRS_*I*_/S2 for improved reconstruction of ET temporal dynamics over irrigated and intensively managed landscapes, confirming the applicability of the proposed multi-source fusion. However, the relatively low spatial contrast found in some VIIRS native and sharpened LST images might impact the accuracy of VIIRS_*I*_/S2 ET retrievals (Bellvert et al. 2020; Guzinski and Nieto 2019; Xue et al. 2021). Future studies targeting improved regression tree algorithms used in DMS may consider adding other bands (e.g., red-edge bands of S2) or incorporating microwave SAR data. The temporal discrepancy between HLS SR and ECOSTRESS LST data is also a challenge because inconsistencies between these inputs may impact both LST sharpening and ET retrievals (Anderson et al. 2021; Hoffmann et al. 2016), especially over landscapes with rapidly changing phenology or moisture status. Future studies targeting daily SR data based on fusion approach may effectively reduce temporal discrepancies and therefore improve ECOSTRESS ET estimates. In addition, differences in pixel footprints from different sensors at varying view angles can also affect LST sharpening, ET disaggregation, data fusion and the consistency of ET time series at fine spatial resolution. This is a challenging issue for all multi-sensor data fusion and applications, especially at field to sub-field scales.

Although multi-source data fusion can partially compensate for the current lack of satellite TIR data with the required resolution, additional direct high-resolution TIR observations to complement the existing capabilities of current satellites are still desirable. A few on-going and future satellite missions with TIR sensors at sub-field resolution, including the Surface Biology and Geology (SBG; Cawse-Nicholson et al. 2021), Land Surface Temperature Monitoring (LSTM; Koetz et al. 2018) and Thermal infrared Imaging Satellite for High-resolution Natural resource Assessment (TRISHNA; Lagouarde et al. 2018) missions will provide new data sources for advancing ET monitoring for agricultural water management. There are also spectral-based approaches using shortwave-infrared bands on Sentinel-2 that have a 5-day return interval and potential to contribute to increased frequency of daily ET (D’Urso et al. 2021). Consistency and compatibility of ET retrievals from different data sources are important for the multi-source ET modeling system. In ALEXI/DisALEXI, these issues are addressed in part by constraining each 30-m ET retrieval by a common dataset of ALEXI ET at a coarser scale. For other modeling platforms, a data harmonization approach or data screening scheme may be needed before using multiple sources in a data fusion system.

### Impact of sampling frequency

In this study, the value of multi-source integration was investigated over agricultural target sites in California, many of which have relatively low levels of cloud cover and optimal clear-sky acquisition frequency. Future work will assess the proposed framework over diverse sites sampling a broader range of cloud cover climatology, including the more humid Central and Eastern United States where there is even stronger demand for extra sampling to complement Landsat (Sun et al. 2017; Yang et al. 2018). These sites may show a more marked statistical improvement in ET timeseries retrieval with multi-source fusion.

With several new and proposed medium-resolution thermal imaging missions on the horizon, the question of sufficient collective revisit frequency for applications including agricultural water management must be addressed. Previous studies suggested that revisit period of four (Anderson et al. 2012; Guillevic et al. 2019) or five (Alfieri et al. 2017; Mercury et al. 2012) days would be sufficient for accurate ET estimation. Using the high-frequency set of multi-source California ET retrievals developed in this study as a baseline, we conducted a similar test on the revisit frequency impact on the accuracy of the fused daily ET.

We started with a 4-day time interval, and randomly selected one 30-m ET sample for each 4-day period if it is available. The selected samples were then interpolated using 500-m VIIRS_*M*_ ET as a scaling flux to produce daily ET timeseries. In this exercise, we used linear interpolation rather than STARFM fusion for computational efficiency, interpolating the ratio of 30-m ET to 500-m VIIRS_*M*_ ET between selected overpasses and then multiplying by daily 500-m to synthesize a daily 30-m timeseries. To ensure robust results, we computed average RMSE with respect to observations from six randomly generated timeseries. Then we repeated this process assuming 7-day, 14-day, and 28-day intervals between overpasses.

**Fig. 17:**
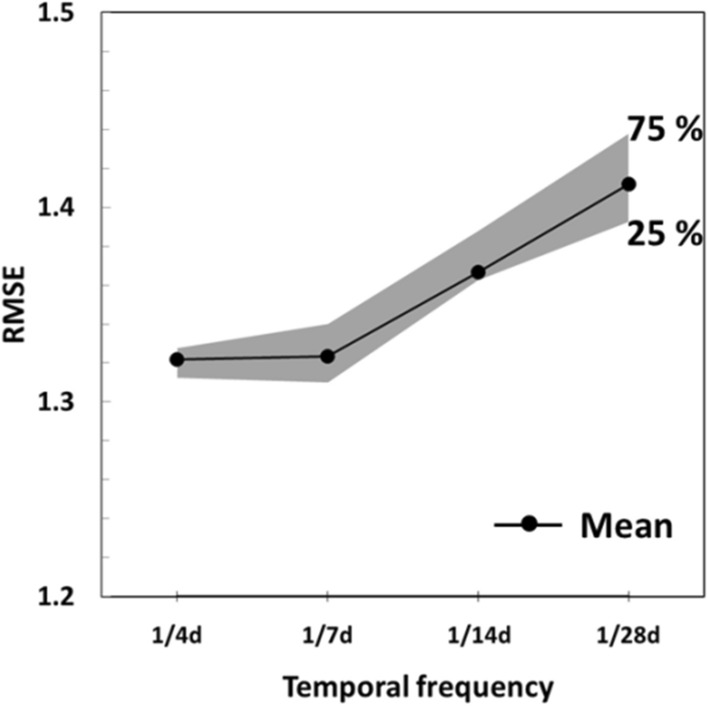
RMSE between measured and interpolated daily ET derived from different revisit scenarios at the RIP760 site in 2020

This experiment was conducted over the RIP760 site for 2020 (Fig. 17). It can be clearly seen that the RMSE increases with respect to the revisit frequency. The results are consistent with Guillevic et al. (2019) who found that a four-day revisit provides a significant improvement over a 16-day revisit for ET monitoring and that the ability to capture rapid changes in ET was significantly reduced for revisit frequency lower than eight days. As pointed out by Lagouarde et al. (2013), only a one-day return interval can provide one cloud-free observation every five days on average in Europe. Overall, satellite TIR observations with high spatiotemporal resolution are highly desired in many agricultural applications.

## Conclusion

In this study, we evaluated the capability of combining multi-source remote sensing data for mapping daily ET at 30-m resolution over six flux tower sites in the Central Valley of California and Northern Coast for 2018–2020 to investigate the utility for informing agricultural water management practices and detecting crop stress. We first compared the performance of 500-m daily ET timeseries generated using VIIRS_*M*_ and MODIS LST and SR inputs, and found comparable or improved accuracy, suggesting that VIIRS_*M*_ can effectively replace MODIS as the fusion backbone of the multi-source ET modeling system as MODIS approaches end of life. The utility of ECOSTRESS and VIIRS I-5 band LST data together with the HLS SR dataset in generating 30-m ET was explored and the results demonstrated that these sources provide comparable accuracy to Landsat ET based on both qualitative and quantitative measures, in comparison with flux tower measurements.

With additional temporal sampling from DisALEXI-ECOSTRESS and DisALEXI-VIIRS_*I*_/S2, the daily 30-m ET estimates obtained from Landsat + ECOSTRESS + VIIRS_*I*_/S2 fusion generally outperformed the Landsat-only fusion for most of the sites, especially over an alfalfa site that exhibits significant temporal variability in ET over the growing season. Fused timeseries based on the three thermal data sources combined better captured temporal dynamics in ET over multiple growing seasons in comparison with Landsat-only fusion. The added value from extra sampling becomes larger in real-time applications, which could be further amplified when the data latency is considered.

We also demonstrated the utility of multi-source fused daily ET timeseries for remotely detecting water stress signals used to manage regulated deficit irrigation in vineyards. Anomalous water use in two vineyard blocks in 2020 relative to 2018–2019 due to changes in vine management practices as well as drought is well represented by the fused monthly ET and *f*_RET_ anomalies. The changes in moisture conditions associated with land cover changes over surrounding fields were also revealed by the *f*_RET_ products. Improved detection of anomalous water use and stress could help the managers to adapt irrigation applications in real time and therefore improve the quality and yield in grape production.

## Data Availability

The data analyzed during the current study are available from the corresponding author upon reasonable request.
